# Hepatitis B Virus Infection and Risk Factors Among Pregnant Women in Healthcare Facilities in West Africa: A Systematic Review and Meta‐Analysis

**DOI:** 10.1155/bmri/3975525

**Published:** 2026-03-24

**Authors:** Fatimata Hassane, Adamou Lagare, Abdourahamane Yacouba, Donatien Serge Mbaga, Larwanou Harouna Magagi, Moussa Issa, Haboubacar E.Y. Moussa, Arnol Bowo-Ngandji, Mahamadou Doutchi, Alkassoum Salifou Ibrahim, Haoua Sabo Seini, Mamadou Saidou

**Affiliations:** ^1^ Centre de Recherche Médicale et Sanitaire (CERMES), Niamey, Niger, cermes.net; ^2^ Faculté Des Sciences de la Santé, Université Abdou Moumouni, Niamey, Niger, uam.refer.ne; ^3^ Department of Biomedical Sciences, University of Bertoua, Bertoua, Cameroon, univ-bertoua.cm; ^4^ University Sechenov, Moscow, Russia; ^5^ Department of Microbiology, University of Yaounde, Yaounde, Cameroon; ^6^ University Andre Salifou, Zinder, Niger

**Keywords:** hepatitis B, pregnant women, risks factors, West Africa

## Abstract

**Background:**

Hepatitis B virus (HBV) infection remains a critical global health issue, affecting over 254 million individuals and causing approximately 1.2 million new infections annually. This systematic review and meta‐analysis is aimed at estimating the pooled prevalence of HBV infection and identifying associated risk factors among pregnant women in West African countries.

**Methods:**

We conducted a systematic review and meta‐analysis following the Preferred Reporting Items for Systematic Reviews and Meta‐Analyses (PRISMA) guidelines. We searched Ovid Medline, Ovid Global Health, Ovid Embase, Web of Science, African Journals Online (AJOL), and African Index Medicus for studies on HBV infection in pregnant women in West African countries. We assessed the methodological quality of the included studies using the Hoy et al. tool. The random‐effects model was used to calculate the pooled prevalence of HBV infection, whereas the I^2^ statistic quantified heterogeneity. Egger′s test and funnel plots evaluated publication bias.

**Results:**

This analysis included 138 studies from 11 West African countries. The pooled prevalence of HBV infection, based on HBsAg seropositivity, was 8.0% (95% CI: 7.3–8.6), with significant heterogeneity between studies (I^2^ = 96.8*%*, *p* < 0.001). The prevalence of HBeAg was 15% (95% CI: 10.5–19.9, I^2^ = 79.3*%*, *p* < 0.01). HBV prevalence did not significantly differ between pregnant women with a history of blood transfusion or dental care compared with those without these exposures. Body tattoos were associated with HBV infection, with an odds ratio of 2.28 (95% CI: 1.08–4.79).

**Conclusion:**

This systematic review reveals a substantial prevalence of HBV infection among pregnant women in West Africa, underscoring the urgent need to enhance prevention efforts. Improved access to diagnostic testing and vaccination remains essential to achieving HBV elimination in this population.

## 1. Background

Hepatitis B virus (HBV) infection poses a significant global health challenge, affecting over 254 million individuals and causing 1.2 million new infections annually [1]. HBV accounts for 4.1% of global chronic prevalence across all age groups and contributes to more than 47% of deaths related to viral hepatitis [2, 3]. This infection also causes approximately 22.36% of deaths linked to liver cirrhosis and other chronic liver diseases [4]. The burden of HBV is particularly pronounced in the Western Pacific and African regions, with 97 and 65 million people chronically infected, respectively [5]. Prevalence rates reach 6.2% in the Western Pacific and 6.1% in the African region, underscoring the disproportionate impact in these areas [6]. Due to its global impact, HBV is often referred to as a “silent killer” [7]. HBV transmission from pregnant women to their newborns remains a critical concern, representing a major route of neonatal infection [8]. Women infected during pregnancy face a 70%–90% likelihood of transmitting the virus to their children, with 85%–90% of these infants becoming chronic carriers [9]. Viral hepatitis during pregnancy can increase the incidence of preterm birth (PTB), increase fetal growth, and reduce pregnancy hypertensive disorders [10]. Vertical transmission exposes infants to long‐term risks, including chronic hepatitis, cirrhosis, and liver cancer [11].

Despite the severity of this burden, perinatal transmission of HBV is largely preventable [12]. Administering the hepatitis B vaccine at birth (HepB‐BD) reduces transmission risks to 20%–30% in infants born to HBeAg‐positive mothers and to less than 0.5% in those born to HBeAg‐negative mothers [13]. This preventive measure is crucial in the global fight against HBV, offering hope for reducing the burden of HBV‐related diseases in future generations. However, access to HBV screening and vaccination remains inequitable, particularly in low‐ and middle‐income countries, where less than 1% of chronic HBV patients are aware of their condition [14]. Barriers such as limited healthcare infrastructure, inadequate diagnostic tools, insufficient surveillance, and weak political commitment impede progress. According to the global strategy developed by WHO to eliminate hepatitis B by 2030, preventing mother‐to‐child transmission becomes a key intervention to achieve this goal [15]. West Africa bears a disproportionate burden, with prevalence rates exceeding the global average [16]. An estimated 8.58% of pregnant women in the region are infected, with country‐specific rates varying from 4.68% in Ghana to 9.73% in Nigeria [16]. Previous studies, limited in scope and timeframe, fail to provide a comprehensive overview of HBV infection in West Africa. This systematic review and meta‐analysis is aimed at estimating the aggregated prevalence of HBV infection and identifying associated risk factors among pregnant women in West African countries.

## 2. Methods

### 2.1. Protocol Registration and Review Design

This study was performed and reported using the Preferred Reporting Items for Systematic Reviews and Meta‐Analysis (PRISMA) guidelines (Table S1) [17, 18]. The protocol was registered in the PROSPERO database under the number: CRD42024499335.

### 2.2. Search Strategy

We developed search strategies for Medline (Ovid), Embase (Ovid), Global Health (Ovid), Web of Science, African Journal Online (AJOL) and African Index Medicus, targeting articles published from the inception of these databases to April 3, 2024 (Table S2). The following search terms and their variations were used: hepatitis B and pregnant women, followed by the names of the 16 West African countries [19]. In addition to database searches, we manually screened reference lists for relevant articles. Only studies published in English or French were included, with no restrictions on publication date.

### 2.3. Eligibility Criteria

Two reviewers (F.H. and H.E.Y.M.) checked the titles and abstracts of the articles found in the bibliographic databases, after removing duplicates. They selected studies meeting the following criteria: observational studies (case‐control, cross‐sectional, or cohort) that addressed the prevalence and risk factors among pregnant women in a West African country, articles that detect HBsAg, HBeAg, or HBV DNA in pregnant women. We excluded studies that were not conducted in West Africa, were duplicates, or lacked abstracts or complete texts. We also excluded studies that were not written in English or French and those that did not provide relevant or sufficient data.

### 2.4. Study Selection

Two independent reviewers, F.H. and H.E.Y.M., assessed the potential relevance of studies based on predefined inclusion and exclusion criteria. We initially screened the titles and abstracts of all publications, selecting those that appeared to meet the criteria. Subsequently, the full texts of these selected studies were carefully examined to determine their final inclusion in the review. Any disagreements or inconsistencies between the reviewers were resolved through discussion and consensus. If consensus could not be reached, a third reviewer was consulted to provide a resolution.

### 2.5. Data Extraction and Risk of Bias Evaluation

F.H. and D.S.M independently reviewed the data from the chosen studies. These data were gathered online using Google Forms and organized in a Microsoft Excel spreadsheet. For each study, we recorded the first author′s name, publication year, reason for exclusion (if applicable), study design, country, sampling method, study setting, sample collection timing, countries involved, geographic regions, number of participants screened, and number of participants infected with HBV. The quality assessment of the studies that met the inclusion criteria was rated for methodological quality by two investigators (F.H. and D.S.M) independently. Quality assessment of the included studies was done by using the Hoy et al. [20] tools (Table S3). Any disagreements were settled verbally, and consensus was obtained.

### 2.6. Data Analysis

This statistical analysis used R software (Version 4.0.3) with the meta (Version 4.18‐2) and metafor (Version 3.0‐2) packages [21, 22] to calculate the pooled percentage and 95% confidence interval (CI) using a random‐effects model [23]. The analysis stratified results by geographical, regional location, vaccination period, HBV diagnostic method, gestational age, parity, and gravidity. Heterogeneity was evaluated using the I^2^ statistic, with significant heterogeneity defined as I^2^ values > 50% [24–26]. We performed univariable meta‐regression analyses to explore the potential impact of study‐level covariates on HBV prevalence among pregnant women. Covariates included year of publication, study type, study design, country, vaccination period, and HBV diagnostic method. For categorical covariates, the most frequent category was set as the reference level. Random‐effects meta‐regression models were fitted using the restricted maximum likelihood (REML) method. Bubble plots were generated to visualize the relationship between each covariate and prevalence, with point sizes proportional to study weights. Regression tests investigated publication bias.

## 3. Results

### 3.1. Search Strategy

**Figure 1:**
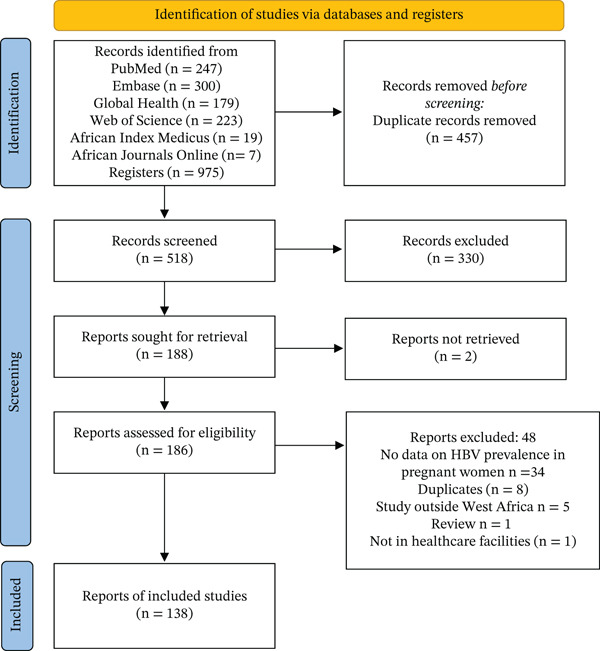
PRISMA diagram showing the selection of studies.

We searched six databases, including Medline (Ovid), Embase (Ovid), Global Health (Ovid), Web of Science, AJOL, and African Index Medicus, identifying 975 records. After removing duplicates (457) and excluding the irrelevant articles (330), 188 articles were examined. Following a thorough evaluation of the full text, 138 articles were deemed relevant and included in this synthesis (Figure 1).

### 3.2. Characteristics of Included Studies

We gathered published data from 11 countries of West Africa, with more than half of the data originating from Nigeria (56.52%) and representation as follows: Benin (2.17%), Burkina Faso (11.59%), Gambia (1.45%), Ghana (15.94%), Ivory Coast (2.17%), Mali (3.62%), Mauritania (0.72%), Niger (0.72%), Senegal (2.17%), and Sierra Leone (2.90%). The characteristics of the included studies are summarized in Table S4. The study period of the included studies ranged from 1977 to 2023. A total of 129 of the 138 studies (93.48%) were cross‐sectional studies, 6 were cohort studies, and 3 were case‐control studies. All 138 studies were conducted in healthcare facilities. The studies were distributed among low‐income countries (20.29%) and lower middle‐income countries (79.71%). The presence of HBV DNA was reported in 16 studies. HBsAg status was reported in 129 studies and 33 studies reported both HBsAg and HBeAg status (Table 1). HBV infection was detected in 56.83% of cases by the ELISA method, in 15.11% of cases using the lateral flow chromatographic immunoassay, and in 9.35% of cases using rapid diagnostic tests. Other detection tests used for HBV detection included the following: agglutination tests, chemiluminescence assays, enzyme immunoassays, radioimmunoassays, and PCR. The studies reviewed had a low 66.42% and moderate risk of bias 33.58% (Table S5). No cases of death were reported in the examined studies.

**Table 1:** Summary of meta‐analysis results for estimates of HBV prevalence in West Africa.

	Prevalence. % (95% CI)	*N* data points	*N* participants	^a^I^2^ (95% CI)	*p* Egger test
HBsAg	8 [7.3–8.6]	129	2928715	96.8 [96.5–97.1]	0.58
HBeAg	15 [10.5–19.9]	33	1603	79.3 [71.5–85]	0.012
DNA	7.6 [5.9–9.4]	16	32808	96.5 [95.4–97.3]	0.010

Abbreviations: 95% CI, 95% confidence interval; N, number; NA, not applicable.

^a^I^2^ describes the proportion of total variation in study estimates that is due to heterogeneity, a value > 50% indicates the presence of heterogeneity.

### 3.3. Prevalence of Hepatitis B (HBsAg) Among Pregnant Women in West Africa

**Figure 2:**
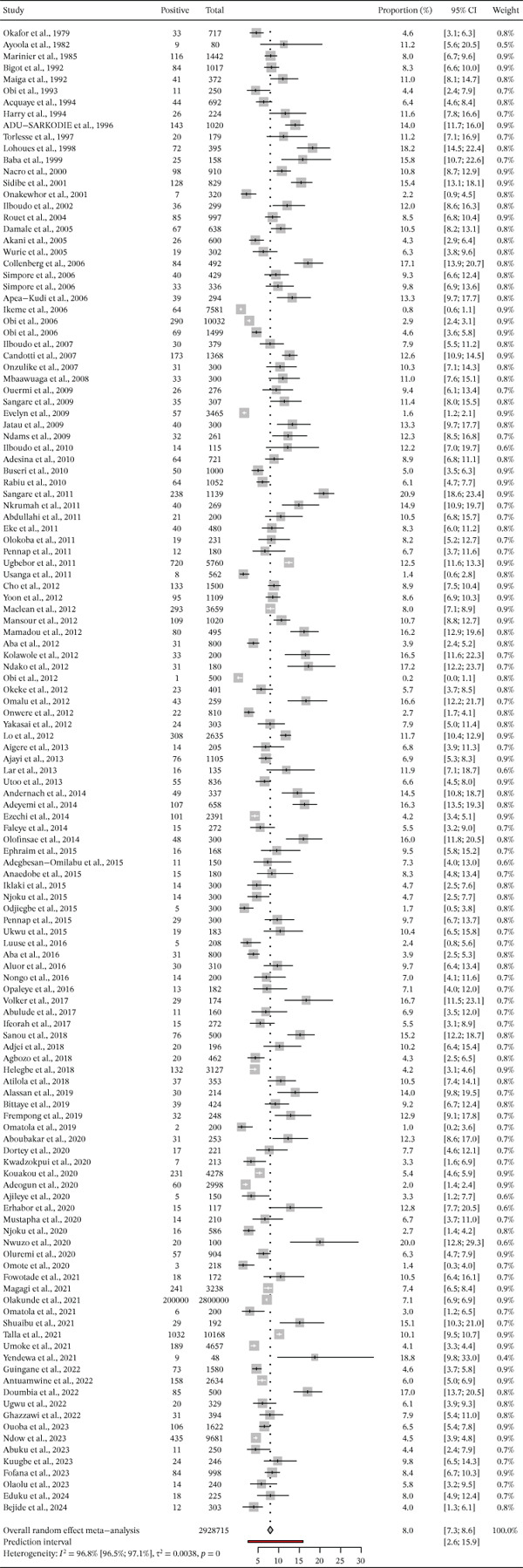
Forest plot of country‐specific hepatitis B (HBsAg) prevalence among pregnant women in West Africa.

**Figure 3:**
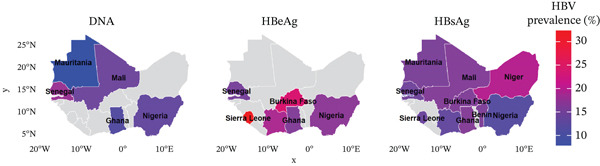
Geographical distribution of hepatitis B prevalence rates among pregnant women in West Africa.

The prevalence of hepatitis B in the 129 studies included in the meta‐analysis varied between 6.5% (95% CI: 2.6, 11.9) and 16.2% (95% CI: 13.0, 19.7). The overall prevalence of HBV infection among pregnant women in West Africa was 8.0% (95% CI: 7.3, 8.6) based on the random‐effect model analysis (Table 1). The heterogeneity test showed high heterogeneity, I^2^ = 96.8*%* and *p* < 0.001. According to the countries, the prevalence rates of HBV were 11%, 11.2%, 6.5%, 8.5.%, 10%, 11.6%, 10.7%, 16.2%, 6.6%, 9.8%, and 9.2% in Benin, Burkina Faso, Gambia, Ghana, Ivory Coast, Mali, Mauritania, Niger, Nigeria, Senegal, and Sierra Leone, respectively (Figures 2 and 3).

### 3.4. Prevalence of HBeAg

**Figure 4:**
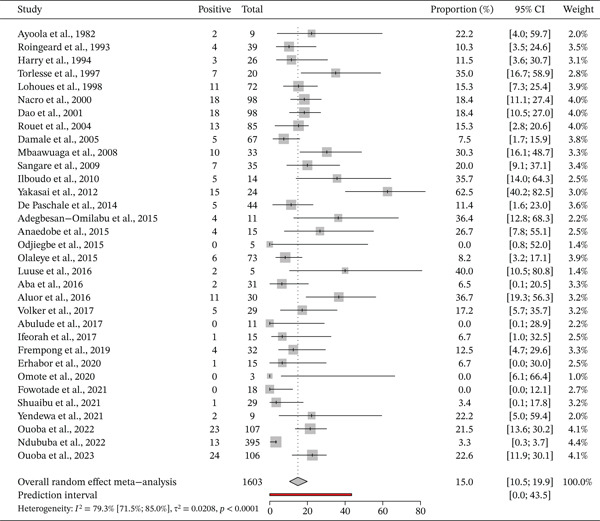
Forest plot of country‐specific hepatitis B (HBeAg) prevalence among pregnant women in West Africa.

Thirty‐three studies with a total sample of 1603 reported the prevalence of HBeAg among pregnant women infected with HBV. The prevalence varied from 10.3% to 30.8% (Figure 3). The overall prevalence of HBeAg in the 33 studies was 15% (95% CI = 10.5*%*–19.9*%*, I^2^ = 79.3*%*, *p* < 0.01) (Figure 4).

### 3.5. Prevalence of HBV DNA

**Figure 5:**
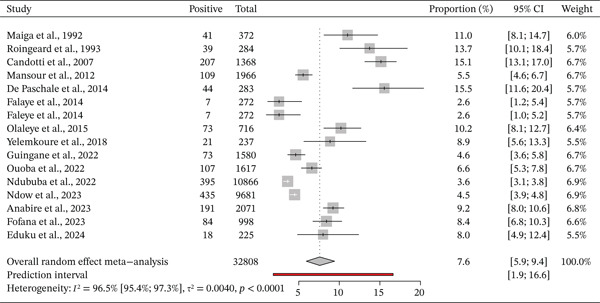
Forest plot of country‐specific hepatitis B (DNA) prevalence among pregnant women in West Africa.

Overall, 16 studies with 10 HBs Ag positive patients reported on the HBV DNA. The prevalence of HBV infection DNA in West Africa varies from 4.5% to 13.7% depending on the country (Figure 3). The pooled prevalence of HBV DNA from the studies included in the meta‐analysis is 7.6% (95% CI: 5.9, 9.4). The heterogeneity test showed high heterogeneity, I^2^ = 96.5*%* and *p* < 0.0001 (Figure 5). Genotype E was the most common HBV genotype reported.

### 3.6. Subgroup Analysis of Prevalence of HBV in Pregnant Women in West Africa

The overall prevalence of HBs Ag infection in pregnant women from low‐income countries was 10.7% (95% CI: 8.7, 12.9), whereas in lower middle‐income countries, it was 7.7% (95% CI: 6.9, 8.5) (Table 2). By vaccination period, the pooled prevalence was 7.8% (95% CI: 6.0, 9.8) for studies conducted in transition period (2000–2010), 7.3% (95% CI: 6.6, 8.1) in the postvaccination period, and 9.9% (95% CI: 6.4, 14.0) in the prevaccination period. According to HBV diagnostic methods, prevalence estimates ranged from 5.7% (95% CI: 4.7, 6.9) using lateral flow chromatography immunoassay to 11.4% (95% CI: 5.2, 19.7) using radioimmunoassay. Among pregnant women in the third trimester, the pooled prevalence of HBV was 8.2% (95% CI: 5.6, 11.2), 11% (95% CI: 6.4, 16.6) in multiparous women, and 11.3% (95% CI: 7.1, 16.4) in multigravida women.

**Table 2:** Subgroup analyses of prevalence of HBV in pregnant women in West Africa.

	Prevalence (%) [95% CI]	*N*data points	*N*participants	*p*difference subtypes
**Countries**				< 0.001
Benin	11 [7.4–15.2]	3	1484	
Burkina Faso	11.2 [8.4–14.4]	14	8721	
Gambia	6.5 [2.6–11.9]	2	10,105	
Ghana	8.5 [6.9–10.3]	21	15,262	
Ivory Coast	10 [4.8–16.7]	3	5670	
Mali	11.6 [8.2–15.6]	5	6358	
Mauritania	10.7 [8.9–12.7]	1	1020	
Niger	16.2 [13–19.5]	1	495	
Nigeria	6.6 [5.8–7.5]	73	2,874,600	
Senegal	9.8 [6.6–13.7]	2	4077	
Sierra Leone	9.2 [5.9–13]	4	923	

**World Bank income groups**				0.006
Low‐income countries	10.7 [8.7–12.9]	25	26,265	
Lower middle‐income countries	7.7 [6.9–8.5]	104	2,902,780	

**Vaccination period**				0.394
Transition period (2000–2010)	7.8 [6–9.8]	36	50266	
Postvaccination (post‐2010)	7.3 [6.6–8.1]	49	2,852,537	
Prevaccination (pre‐2000)	9.9 [6.4–14]	6	4473	

**HBV diagnostic method**				0.002
Chemiluminescence immunoassay	6.5 [5.4–7.8]	1	1622	
ELISA	9.4 [7.8–11.2]	77	57,878	
Lateral flow chromatography immunoassay	5.7 [4.7–6.9]	34	56,642	
Latex agglutination test	6.4 [2.5–11.9]	5	4858	
Radioimmunoassay	11.4 [5.2–19.7]	2	2271	

**Gestational age**				0.724
First trimester	5.3 [2.3–9.3]	17	1294	
Second trimester	6.9 [4.8–9.3]	20	4851	
Third trimester	8.2 [5.6–11.2]	21	3900	

**Parity**				0.015
Multiparous	11 [6.4–16.6]	21	9350	
Nulliparous	3.8 [1.9–6.4]	7	1208	
Primiparous	8.1 [4.3–12.8]	20	6599	

**Gravidity**				0.377
Multigravidae	11.3 [7.1–16.4]	9	3748	
Primigravidae	8.6 [5.7–11.9]	9	4034	

In the meta‐regression analysis, year of publication, study type, study design, vaccination period, and most country‐level covariates were not significantly associated with HBsAg prevalence (Table S6). A statistically significant association was observed in Burkina Faso compared with Nigeria (*p* = 0.008). Regarding diagnostic methods, studies using lateral flow tests showed significantly lower prevalence estimates than those using ELISA (*p* = 0.002).

### 3.7. Factors Associated With HBV Infection

#### 3.7.1. History of Blood Association

**Figure 6:**
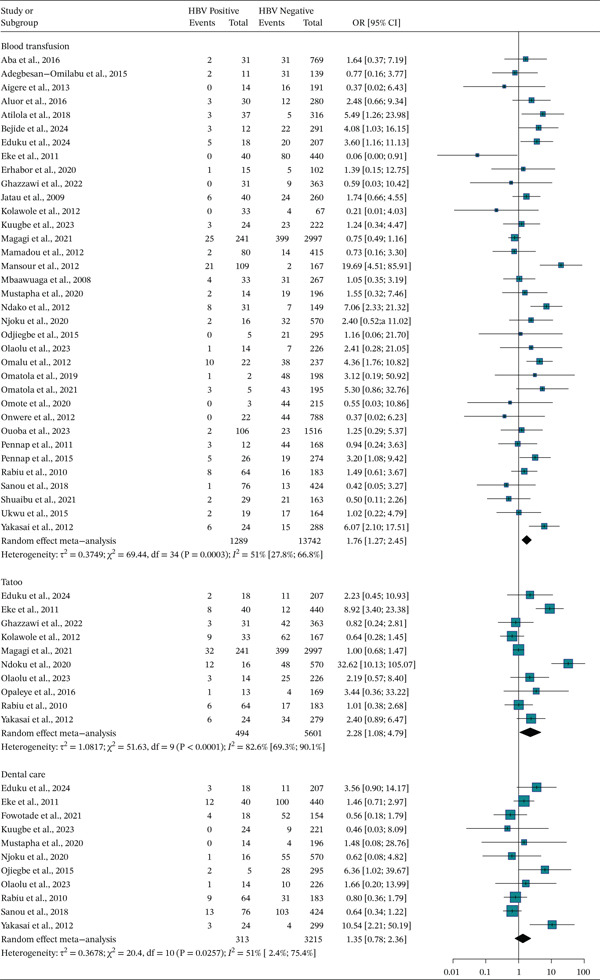
Forest plot of risk factors associated with HBV infection: blood transfusion, body tattoo, and dental care history.

Thirty‐five studies, which involve 13,742 pregnant women, were included in this category of meta‐analysis. Eight of the included studies showed the presence of association of blood transfusion with a higher risk of HBV infection (Figure 6). The meta‐analysis showed a weak association between blood transfusion and HBV infection, OR = 1.76 (95% CI: 1.27; 2.45). The heterogeneity test showed statistical evidence of heterogeneity, I^2^ = 51*%* and *p* value = 0.0003.

#### 3.7.2. Body Tattoo

Ten studies, involving 5601 pregnant women, were included to determine the association of history of body tattoo and HBV infection. The pooled meta‐analysis showed a strong association between body tattoo and HBV infection OR = 2.28 (95% CI: 1.08; 4.79). The heterogeneity test showed evidence of high heterogeneity, I^2^ = 82.6*%* and *p* value ≤ 0.001 (Figure 6).

#### 3.7.3. Dental Care

A total of 11 studies were included in the meta‐analysis to determine the association between history of dental care and HBV infection. There is no significant association between the history of dental care and HBV infection OR = 1.08 (95% CI: 0.78; 2.36). Heterogeneity test showed evidence of high heterogeneity, I^2^ = 51*%* and *p* value = 0.0257 (Figure 6).

The available genotype data show that genotype E was the most frequently detected HBV genotype across studies in West Africa (Table S7). In Ghana, genotype E accounted for the majority of cases (175/191 in Anabire et al. [27]; 69/70 in Candotti et al. 2007), with smaller proportions of mixed genotypes including A and D. In Nigeria, only genotype E was reported. In Burkina Faso, genotype E was also the most common (37/63), followed by genotype A (23/63).

## 4. Discussion

This systematic review and meta‐analysis revealed a high prevalence of HBV infection among pregnant women in West Africa. The pooled prevalence of HBsAg seropositivity was 8.0% (95% CI: 7.3%–8.6%), indicating a high level of endemicity based on WHO classification [28]. Country‐specific prevalence rates ranged from 6.5% in Gambia to 16.2% in Niger, with Benin, Burkina Faso, Ghana, Ivory Coast, Mali, Mauritania, Nigeria, Senegal, and Sierra Leone also exhibiting high prevalence rates exceeding 7%. The pooled prevalence in low‐income countries was higher (10.7%, 95% CI: 8.7–12.9) than in lower middle‐income countries (7.7%, 95% CI: 6.9%–8.5%). Additionally, the prevalence of HBV DNA was 7.6% (95% CI: 5.9%–9.4%), whereas HBeAg prevalence among HBsAg‐positive pregnant women was 15% (95% CI: 10.5%–19.9%).

The findings of this study align with previous reports from the West African region, including Larebo et al. [16], which reported an HBV prevalence of 8.58% among pregnant women, although the present study includes a more extensive dataset. The prevalence observed in this meta‐analysis is higher than pooled estimates from Nigeria (6.49%) [12] and Ethiopia (5.78%) [29]. However, it remains lower than prevalence rates reported in Kenya (9.3%) [30], Cameroon (9.8%) [31], and cross‐sectional studies in Nigeria (10.2%) [32]. The pooled prevalence of HBV DNA (7.6%) and HBeAg (15%) in this study is consistent with prior studies highlighting the significant infectivity and transmission risks associated with HBeAg positivity.

The variations in HBV prevalence across countries and regions can be attributed to several factors. Sociocultural practices, healthcare access disparities, and the availability of vaccination and screening programs play critical roles in shaping the epidemiology of HBV. For example, the higher prevalence in low‐income countries (10.7%) compared with lower middle‐income countries (7.7%) reflects disparities in healthcare infrastructure and prevention strategies. The results of our review showed that overall HBV prevalence among pregnant women in West Africa remains high, dropping from 9.9% in the prevaccination era to 7.3% in the postvaccination period. Although this downward trend suggests that the introduction of the HBV vaccine into the Expanded Program on Immunization (EPI) in the early 2000s is bearing fruit, prevalence remains firmly at the threshold of high endemicity (≥ 8%) or high intermediate endemicity (5%–7%) according to WHO classification standards [12, 33]. This small decrease in prevalence can be attributed to the fact that many women currently of childbearing age were born before the widespread implementation of the birth dose and full infant immunization schedule, which was adopted by only 15 of the 47 countries in the African region until recent years [34, 35].

Furthermore, the variation in HBV prevalence by diagnostic method showed that the value obtained with the radioimmunoassay technique (11.4%) was twice as high as that obtained with the lateral flow chromatography method (5.7%). These results underline that rapid diagnostic tests, although cost‐effective, could underestimate the true disease burden by as much as half [36, 37].

The HBV genetics data obtained reveal that genotype E is the most frequently detected genotype in the region, being almost exclusive in Nigeria and dominant in Ghana and Burkina Faso. Indeed, genotype E is specific to sub‐Saharan Africa; it is characterized by high viral loads and a lower frequency of HBe antigen positivity in certain cohorts, which may mask the risk of a high rate of vertical transmission [27, 38]. The presence of lower proportions of genotypes A and D in Ghana and Burkina Faso introduces additional complexity, as the different genotypes may respond differently to antiviral therapies and influence long‐term progression to cirrhosis or hepatocellular carcinoma [39]. These findings underline the urgency of adopting more sensitive screening tools and genotype‐specific clinical follow‐up in order to achieve the WHO′s 2030 elimination target of a 90% reduction in new infections [34, 40].

Methodological differences, including the inclusion of hospital‐based versus community‐based studies, may explain the variability in prevalence rates, as hospital‐based studies often reflect a referral bias with higher proportions of severely ill individuals. Behavioral risk factors, such as body tattooing, were significantly associated with HBV infection (OR = 2.28, 95% CI: 1.08–4.79) and are prevalent in this region. In contrast, factors such as dental care did not show significant associations with HBV infection (OR = 1.08, 95% CI: 0.78–2.36). These findings highlight the multifactorial drivers of HBV transmission in West Africa.

This study has several limitations. The high heterogeneity observed across studies, driven by differences in methodology, population characteristics, and publication years, may have influenced the pooled estimates. Additionally, the lack of community‐based studies limits the generalizability of the findings, as many pregnant women may not attend healthcare facilities for antenatal care. Publication bias and the exclusion of non‐English or non‐French articles further constrain the representativeness of the results. Variations in study design and diagnostic tools also contributed to discrepancies, complicating comparisons between studies. Despite these limitations, this meta‐analysis has notable strengths. It employed a robust methodology aligned with PRISMA guidelines and utilized a comprehensive search strategy across multiple databases. By integrating data from numerous studies, this analysis offers precise estimates of HBV prevalence.

This meta‐analysis confirmed the high prevalence of HBV infection among pregnant women in West Africa, with significant heterogeneity across countries. The study highlights the necessity of preventing mother‐to‐child transmission through systematic prenatal screening, antiviral therapy administration during the third trimester for high‐risk pregnancies, and birth‐dose vaccination combined with postexposure prophylaxis for newborns. These measures are essential to interrupt the vertical transmission of HBV and mitigate its long‐term public health burden. To achieve the WHO′s 2030 elimination target for hepatitis B, region‐specific public health interventions are essential to reduce perinatal transmission and the burden of HBV in this vulnerable population. To address the high burden of HBV infection among pregnant women, several targeted recommendations are essential. Integrating HBV screening into routine antenatal care should be prioritized to ensure systematic testing of all pregnant women as part of standard reproductive health services. Strengthening prevention of mother‐to‐child transmission initiatives is critical and includes administering antiviral therapy during the third trimester for high‐risk pregnancies, implementing birth‐dose vaccination, and providing postexposure prophylaxis for newborns. Enhancing health education campaigns is also necessary to raise awareness among pregnant women about HBV transmission, risk factors, and preventive measures, such as avoiding shared sharp objects and unsterilized equipment. Expanding vaccination programs is vital to increase HBV vaccination coverage for both mothers and neonates, particularly in high‐prevalence regions. Further research should be promoted to address gaps in understanding risk factors, regional disparities, and the effectiveness of current prevention strategies. Lastly, collaboration with governments and nongovernmental organizations is crucial to improve access to antiviral therapies and diagnostics while addressing the stigma associated with HBV diagnosis. These comprehensive strategies will contribute significantly to reducing HBV‐related morbidity and mortality, particularly in resource‐limited settings.

## 5. Conclusion

This systematic review reveals a substantial prevalence of HBV infection among pregnant women in West Africa, underscoring the urgent need to enhance prevention efforts. Improved access to diagnostic testing and vaccination remains essential to achieving HBV elimination in this population.

## Author Contributions

Conceptualization: F.H. and A.L.; data curation: D.S.M, H.E.Y.M, and L.H,M.; methodology and field investigations: F.H., D.S.M, A.Y., A.S.Y., and M.I.; formal analysis: M.D. and A.B‐N.; writing: F.H. and A.L.; validation and project administration: A.L., H.S.S., and M.S.

## Funding

No funding was received for this manuscript.

## Disclosure

All authors read and approved the final version of the manuscript.

## Conflicts of Interest

The authors declare no conflicts of interest.

## Supporting Information

Additional supporting information can be found online in the Supporting Information section.

## Supporting information


**Supporting Information 1** Table S1: Preferred Reporting Items for Systematic Reviews and Meta‐Analyses checklist.

**Supporting Information 2** Table S2: Search strategy.

**Supporting Information 3** Table S3: Items for risk of bias assessment.

**Supporting Information 4** Table S4: Individual characteristics of included studies.

**Supporting Information 5** Table S5: Risk of bias assessment.

**Supporting Information 6** Table S6: Meta‐regression analyses of prevalence of HBV in pregnant women in West Africa.

**Supporting Information 7** Table S7: Genotypes of HBV in pregnant women in West Africa.

## Data Availability

The data that support the findings of this study are available on request from the corresponding author. The data are not publicly available due to privacy or ethical restrictions.
