# Supplementary material for: Hepatitis B Virus Infection and Risk Factors Among Pregnant Women in Healthcare Facilities in West Africa: A Systematic Review and Meta‐Analysis
Source: Biomed Res Int. 2026 Mar 24;2026:3975525. doi: 10.1155/bmri/3975525 (PMC13140436; doi:10.1155/bmri/3975525)
Supplement: Supplementary file 2 — Supporting Information 2 Table S2: Search strategy. [file BMRI-2026-3975525-s003.docx]

S2 Table: Search strategy

| Database |  | Search (done on April 03, 2024) | Items |
| --- | --- | --- | --- |
| Medline (Ovid) | 1 | exp Hepatitis B virus/ or exp Hepatitis B/ or exp Hepatitis B, Chronic/ or exp Hepatitis B Surface Antigens/ | 80601 |
|  | 2 | (Hepatitis B virus or Hepatitis B or HBV or chronic hepatitis B).mp. | 117900 |
|  | 3 | exp Africa, Western/ or exp Benin/ or exp Burkina Faso/ or exp Cabo Verde/ or exp Cote d'Ivoire/ or exp Gambia/ or exp Ghana/ or exp Guinea/ or exp Guinea-Bissau/ or exp Liberia/ or exp Mali/ or exp Mauritania/ or exp Niger/ or exp Nigeria/ or exp Senegal/ or exp Sierra Leone/ or exp Togo/ | 79797 |
|  | 4 | (Benin or Burkina Faso or Cabo Verde or Cape Verde or Cote d'Ivoire or Gambia or Ghana or Guinea or Guinea Bissau or Guinea-Bissau or Ivory Coast or Liberia or Mali or Mauritania or Niger or Nigeria or Senegal or Sierra Leone or Togo or West Africa or West African or Western Africa or Western African).mp. | 288315 |
|  | 5 | exp Pregnant Women/ or exp Pregnancy/ | 1028229 |
|  | 6 | (Pregnant Women or Pregnancy or Expecting women or Childbearing or Expecting mother).mp. | 1133072 |
|  | 7 | 1 or 2 | 117900 |
|  | 8 | 3 or 4 | 289722 |
|  | 9 | 5 or 6 | 1149030 |
|  | 10 | 7 and 8 and 9 | 247 |
|  |  |  |  |
| Embase (Ovid) | 1 | exp Hepatitis B virus/ or exp Hepatitis B/ | 160205 |
|  | 2 | (Hepatitis B virus or Hepatitis B or HBV or chronic hepatitis B).mp. | 208906 |
|  | 3 | exp Benin/ or exp Burkina Faso/ or exp Cape Verde/ or exp Cote d'Ivoire/ or exp Gambia/ or exp Ghana/ or exp Guinea/ or exp Guinea-Bissau/ or exp Liberia/ or exp Mali/ or exp Mauritania/ or exp Niger/ or exp Nigeria/ or exp Senegal/ or exp Sierra Leone/ or exp Togo/ | 102968 |
|  | 4 | (Benin or Burkina Faso or Cabo Verde or Cape Verde or Cote d'Ivoire or Gambia or Ghana or Guinea or Guinea Bissau or Guinea-Bissau or Ivory Coast or Liberia or Mali or Mauritania or Niger or Nigeria or Senegal or Sierra Leone or Togo or West Africa or West African or Western Africa or Western African).mp. | 357453 |
|  | 5 | exp Pregnant Women/ or exp Pregnancy/ | 995186 |
|  | 6 | (Pregnant Women or Pregnancy or Expecting women or Childbearing or Expecting mother).mp. | 1228695 |
|  | 7 | 1 or 2 | 208906 |
|  | 8 | 3 or 4 | 357453 |
|  | 9 | 5 or 6 | 1241302 |
|  | 10 | 7 and 8 and 9 | 300 |
|  |  |  |  |
| Global Health (Ovid) | 1 | (Hepatitis B virus or Hepatitis B or HBV or chronic hepatitis B).mp. | 45624 |
|  | 2 | (Benin or Burkina Faso or Cabo Verde or Cape Verde or Cote d'Ivoire or Gambia or Ghana or Guinea or Guinea Bissau or Guinea-Bissau or Ivory Coast or Liberia or Mali or Mauritania or Niger or Nigeria or Senegal or Sierra Leone or Togo or West Africa or West African or Western Africa or Western African).mp. | 114668 |
|  | 3 | (Pregnant Women or Pregnancy or Expecting women or Childbearing or Expecting mother).mp. | 149903 |
|  | 4 | 1 and 2 and 3 | 179 |
|  |  |  |  |
| Web of Science | 1 | All field = ((Hepatitis B virus or Hepatitis B or HBV or chronic hepatitis B) AND (Benin or Burkina Faso or Cabo Verde or Cape Verde or Cote d'Ivoire or Gambia or Ghana or Guinea or Guinea Bissau or Guinea-Bissau or Ivory Coast or Liberia or Mali or Mauritania or Niger or Nigeria or Senegal or Sierra Leone or Togo or West Africa or West African or Western Africa or Western African)) AND (Pregnant Women or Pregnancy or Expecting women or Childbearing or Expecting mother) | 223 |
|  |  |  |  |
| Africa Index Medicus | 1 | (Hepatitis B virus or HBV) AND (Pregnant Women) | 19 |
| Total |  |  | 968 |
| Duplicates |  |  | 450 |
| Screened |  |  | 518 |
