# Supplementary material for: Hepatitis B Virus Infection and Risk Factors Among Pregnant Women in Healthcare Facilities in West Africa: A Systematic Review and Meta‐Analysis
Source: Biomed Res Int. 2026 Mar 24;2026:3975525. doi: 10.1155/bmri/3975525 (PMC13140436; doi:10.1155/bmri/3975525)
Supplement: Supplementary file 3 — Supporting Information 3 Table S3: Items for risk of bias assessment. [file BMRI-2026-3975525-s005.docx]

S3 Table: Items for risk of bias assessment

| Hoy et al. tool for cross sectional studies | Yes (1)/No (0) |
| --- | --- |
| 1. Was the study’s target population a close representation of the national population in relation to HBV prevalence in pregnant women? | 1 |
| 2. Was the sampling frame a true or close representation of the target population? | 1 |
| 3. Was some form of random selection used to select the sample, OR was acensus undertaken? | 1 |
| 4. Were data collected directly from the subjects (as opposed to a proxy)? |  |
| 5. Was an acceptable inclusion criteria definition used in the study? |  |
| 6. Did the author calculate and respect the expected sample size? |  |
| 7. Was the HBV detection assay shown to have reliability and validity? | 1 |
| 8. Was the same mode of data collection used for all subjects? | 1 |
| 9. Was the length of the study period > or = 1 year? | 1 |
| 10. Were the numerator(s) and denominator(s) for the HBV data in pregnant women appropriate? | 1 |
| Total score | 10 |
| Interpretation of the risk of bias tool   - 7-10: Low risk of bias - 4-6: Moderate risk of bias - 0-3: High risk of bias |  |

Modified from: Hoy D, Brooks P, Woolf A, Blyth F, March L, Bain C, et al. Assessing risk of bias in prevalence studies: modification of an existing tool and evidence of interrater agreement. J Clin Epidemiol. 2012;65: 934–939. doi:10.1016/j.jclinepi.2011.11.014
