# Supplementary material for: Hepatitis B Virus Infection and Risk Factors Among Pregnant Women in Healthcare Facilities in West Africa: A Systematic Review and Meta‐Analysis
Source: Biomed Res Int. 2026 Mar 24;2026:3975525. doi: 10.1155/bmri/3975525 (PMC13140436; doi:10.1155/bmri/3975525)
Supplement: Supplementary file 4 — Supporting Information 4 Table S4: Individual characteristics of included studies. [file BMRI-2026-3975525-s002.docx]

S4 Table: Individual characteristics of included studies

| **Authors** | **Study Design** | **Setting** | **Countries** | **Study period** | **HBV diagnostic method** | **Sample types** |
| --- | --- | --- | --- | --- | --- | --- |
| Aba et al., 2012 [1] | Cross sectional; Prospective | Hospital-based | Nigeria | Not specified | ELISA | Blood |
| Aba et al., 2016 [2] | Cross sectional; Prospective | Hospital-based | Nigeria | Aug/2011-Dec/2011 | Lateral flow chromatographic immunoassay | Blood |
| Abdullahi et al., 2011 [3] | Cross sectional; Prospective | Hospital-based | Nigeria | Jan/2011-Mar/2011 | ELISA | Blood |
| Aboubakar et al., 2020 [4] | Cross sectional; Prospective | Hospital-based | Benin | Jun/2018-Sep/2018 | ELISA | Blood |
| Abuku et al., 2023 [5] | Cross sectional; Retrospective | Hospital-based | Ghana | Jun/2018-Sep/2018 | Lateral flow chromatographic immunoassay | Blood |
| Abulude et al., 2017 [6] | Cross sectional; Prospective | Hospital-based | Nigeria | Not specified | Lateral flow chromatographic immunoassay | Blood |
| Acquaye et al., 1994 [7] | Cross sectional; Prospective | Hospital-based | Ghana | Not specified | ELISA | Blood |
| Adegbesan-Omilabu et al., 2015 [8] | Cross sectional; Prospective | Hospital-based | Nigeria | Not specified | ELISA | Blood |
| Adeogun et al., 2020 [9] | Cross sectional; Prospective | Hospital-based | Nigeria | Not specified | ELISA | Blood |
| Adesina et al., 2010 [10] | Cross sectional; Prospective | Hospital-based | Nigeria | 2006-2008 | ELISA | Blood |
| Adeyemi et al., 2014 [11] | Cross sectional; Prospective | Hospital-based | Nigeria | 2011 | ELISA | Blood |
| Adjei et al., 2018 [12] | Cross sectional; Prospective | Hospital-based | Ghana | 2016 | ELISA | Blood |
| Adu-Sarkodie et al., 1996 [13] | Cross sectional; Prospective | Hospital-based | Ghana | 1992 | ELISA | Blood |
| Agbozo et al., 2018 [14] | Cross sectional; Prospective | Hospital-based | Ghana | Not specified | ELISA | Blood |
| Aigere et al., 2013 [15] | Cross sectional; Prospective | Hospital-based | Nigeria | 2010 | Lateral flow chromatographic immunoassay | Blood |
| Ajayi et al., 2013 [16] | Cross sectional; Prospective | Hospital-based | Nigeria | 2009 | Lateral flow chromatographic immunoassay | Blood |
| Ajileye et al., 2020 [17] | Cross sectional; Prospective | Hospital-based | Nigeria | Not specified | Rapid diagnostic tests | Blood |
| Akani et al., 2005 [18] | Cross sectional; Prospective | Hospital-based | Nigeria | Not specified | ELISA | Blood |
| Alassan et al., 2019 [19] | Cross sectional; Prospective | Hospital-based | Benin | 2017 | ELISA | Blood |
| Aluor et al., 2016 [20] | Cross sectional; Prospective | Hospital-based | Nigeria | 2012-2013 | ELISA | Blood |
| Anabire et al., 2023 [21] | Cross sectional; Prospective | Hospital-based | Ghana | 2016-2017 | PCR | Blood |
| Anaedobe et al., 2015 [22] | Cross sectional; Prospective | Hospital-based | Nigeria | 2013 | ELISA | Blood |
| Andernach et al., 2014 [23] | Cross sectional; Prospective | Hospital-based | Burkina Faso | Not specified | ELISA | Blood |
| Antuamwine et al., 2022 [24] | Cross sectional; Retrospective | Hospital-based | Ghana | 2016-2017 | ELISA | Blood |
| Apea-Kudi et al., 2006 [25] | Cross sectional; Prospective | Hospital-based | Ghana | Nov/2000-Dec/2001 | Agglutination test | Blood |
| Atilola et al., 2018 [26] | Cross sectional; Prospective | Hospital-based | Nigeria | Not specified | ELISA | Blood |
| Ayoola et al., 1982 [27] | Cross sectional; Prospective | Hospital-based | Nigeria | Not specified | Agglutination test | Blood |
| Baba et al., 1999 [28] | Cross sectional; Prospective | Hospital-based | Nigeria | Not specified | ELISA | Blood |
| Bejide et al., 2024 [29] | Cross sectional; Prospective | Hospital-based | Nigeria | Apr/2022-Jun/2022 | ELISA | Blood |
| Bigot et al., 1992 [30] | Cross sectional; Prospective | Hospital-based | Benin | Not specified | ELISA | Blood |
| Bittaye et al., 2019 [31] | Cross sectional; Prospective | Hospital-based | Gambia | May/2015-Jul/2015 | Lateral flow chromatographic immunoassay | Blood |
| Buseri et al., 2010 [32] | Cross sectional; Prospective | Hospital-based | Nigeria | Mar/2008- Feb/2009 | ELISA | Blood |
| Candotti et al., 2007 [33] | Cohort ; Prospective | Hospital-based | Ghana | Not specified | ELISA | Blood |
| Cho et al., 2012 [34] | Cross sectional; Prospective | Hospital-based | Ghana | Dec/2008-Mar/2009 | Lateral flow chromatographic immunoassay | Blood |
| Collenberg et al., 2006 [35] | Cross sectional; Prospective | Hospital-based | Burkina Faso | Jul/2003-Jan/2004 | ELISA | Blood |
| Damale et al., 2005 [36] | Cross sectional; Prospective | Hospital-based | Ghana | Not specified | ELISA | Blood |
| Dao et al., 2001 [37] | Cross sectional; Prospective | Hospital-based | Nigeria | Not specified | ELISA | Blood |
| De Paschale et al., 2014 [38] | Cross sectional; Prospective | Hospital-based | Nigeria | Jul/2001-Sep/2001 | ELISA | Blood |
| Dortey et al., 2020 [39] | Cross sectional; Prospective | Hospital-based | Ghana | Not specified | ELISA | Blood |
| Doumbia et al., 2022 [40] | Cross sectional; Prospective | Hospital-based | Mali | Not specified | ELISA | Blood |
| Eduku et al., 2024 [41] | Cross sectional; Prospective | Hospital-based | Ghana | 2023 | Rapid diagnostic tests | Blood |
| Eke et al., 2011 [42] | Cross sectional; Prospective | Hospital-based | Nigeria | Aug/2023-Oct/2009 | ELISA | Blood |
| Ephraim et al., 2015 [43] | Cross sectional; Prospective | Hospital-based | Ghana | Dec/2012-Fev/2023 | Rapid diagnostic tests | Blood |
| Erhabor et al., 2020 [44] | Cross sectional; Prospective | Hospital-based | Nigeria | Not specified | Lateral flow chromatographic immunoassay | Blood |
| Evelyn et al., 2009 [45] | Cross sectional; Prospective | Hospital-based | Nigeria | Not specified | Agglutination test | Blood |
| Ezechi et al., 2014 [46] | Cross sectional; Prospective | Hospital-based | Nigeria | Dec/2006-Jan2011 | Lateral flow chromatographic immunoassay | Blood |
| Falaye et al., 2014 [47] | Cross sectional; Prospective | Hospital-based | Nigeria | Sep/2012-Jun/2013 | ELISA, PCR | Blood |
| Faleye et al., 2014 [48] | Cross sectional; Prospective | Hospital-based | Nigeria | 2012-2013 | ELISA | Blood |
| Fofana et al., 2023 [49] | Cross sectional; Prospective | Hospital-based | Mali | Not specified | Lateral flow chromatographic immunoassay | Blood |
| Fowotade et al., 2021 [50] | Cross sectional; Prospective | Hospital-based | Nigeria | Nov/2018-Feb/2019 | ELISA | Blood |
| Frempong et al., 2019 [51] | Cross sectional; Prospective | Hospital-based | Ghana | May/2012 -May/2013 | ELISA | Blood |
| Ghazzawi et al., 2022 [52] | Cross sectional; Prospective | Hospital-based | Sierra Leone | Jul/2021-Sep/2021 | Lateral flow chromatographic immunoassay | Blood |
| Guingane et al., 2022 [53] | Cohort ; Prospective | Hospital-based | Burkina Faso | Oct/2014-February/2016 | ELISA | Blood |
| Harry et al., 1994 [54] | Cross sectional; Prospective | Hospital-based | Nigeria | Not specified | Not reported | Blood |
| Helegbe et al., 2018 [55] | Cross sectional; Prospective | Hospital-based | Ghana | Mar/2013-Feb/2015 | Rapid diagnostic tests | Blood |
| Ifeorah et al., 2017 [56] | Cross sectional; Prospective | Hospital-based | Nigeria | Sep/2012-Jun/2013 | ELISA | Blood |
| Ikeme et al., 2006 [57] | Cross sectional; Prospective | Hospital-based | Nigeria | Jan/2000–Dec/2004 | ELISA | Blood |
| Iklaki et al., 2015 [58] | Cross sectional; Prospective | Hospital-based | Nigeria | Nov/2013-Apr/2014 | ELISA | Blood |
| Ilboudo et al., 2002 [59] | Cross sectional; Prospective | Hospital-based | Burkina Faso | Jul/1999-Sep/1999 | ELISA | Blood |
| Ilboudo et al., 2007 [60] | Cross sectional; Prospective | Hospital-based | Burkina Faso | Feb/2005-Mar/2006 | ELISA | Blood |
| Ilboudo et al., 2010 [61] | Cross sectional; Prospective | Hospital-based | Burkina Faso | Jan/2007-Apr/2009 | ELISA | Blood |
| Jatau et al., 2009 [62] | Cross sectional; Prospective | Hospital-based | Nigeria | Not specified | Immunoassay kit | Blood |
| Kolawole et al., 2012 [63] | Cross sectional; Prospective | Hospital-based | Nigeria | Dec/2010-Mar/2011 | ELISA | Blood |
| Kouakou et al., 2020 [64] | Cohort ; Prospective | Hospital-based | Ivory Coast | Dec/2016-Jun/2017 | Lateral flow chromatographic immunoassay | Blood |
| Kuugbe et al., 2023 [65] | Cross sectional; Prospective | Hospital-based | Ghana | Aug/2019-Nov/2019 | Rapid diagnostic tests | Blood |
| Kwadzokpui et al., 2020 [66] | Cross sectional; Prospective | Hospital-based | Ghana | Nov/2018-Jan/2019 | Rapid diagnostic tests | Blood |
| Lar et al., 2013 [67] | Cross sectional; Prospective | Hospital-based | Nigeria | May/2008-Jun/2008 | Rapid diagnostic tests | Blood |
| Lo et al., 2012 [68] | Cross sectional; Prospective | Hospital-based | Senegal | Dec/2006-Nov/2010 | Enzyme immunoassay | Blood |
| Lohoues et al., 1998 [69] | Cohort ; Prospective | Hospital-based | Ivory Coast | Aug/1995-Fev/1996 | ELISA | Blood |
| Luuse et al., 2016 [70] | Cross sectional; Prospective | Hospital-based | Ghana | Mar/2016 -May/2016 | ELISA | Blood |
| Maclean et al., 2012 [71] | Cross sectional; Prospective | Hospital-based | Mali | May/2008-Dec/2009 | Lateral flow chromatographic immunoassay | Blood |
| Magagi et al., 2021 [72] | Cross sectional; Prospective | Hospital-based | Nigeria | Nov/2017-Apr/2018 | Lateral flow chromatographic immunoassay | Blood |
| Maiga et al., 1992 [73] | Cross sectional; Prospective | Hospital-based | Mali | Not specified | Not reported | Not reported |
| Mamadou et al., 2012 [74] | Cross sectional; Prospective | Hospital-based | Niger | 2008 | ELISA | DBS |
| Mansour et al., 2012 [75] | Cross sectional; Prospective | Hospital-based | Mauritania | Dec/2008-Mar/2009 | ELISA | Blood |
| Marinier et al., 1985 [76] | Cohort ; Prospective | Hospital-based | Senegal | Apr/1977-Apr/1980 | Radioimmunoassay | Blood |
| Mbaawuaga et al., 2008 [77] | Cross sectional; Prospective | Hospital-based | Nigeria | Jan/2005-Apr/2005 | ELISA | Blood |
| Mustapha et al., 2020 [78] | Cross sectional; Prospective | Hospital-based | Nigeria | Mar/2008-Apr/2018 | ELISA | Blood |
| Nacro et al., 2000 [79] | Cross sectional; Prospective | Hospital-based | Burkina Faso | Not specified | Not reported | Blood |
| Ndako et al., 2012 [80] | Cross sectional; Prospective | Hospital-based | Nigeria | Not specified | ELISA | Blood |
| Ndams et al., 2009 [81] | Cross sectional; Prospective | Hospital-based | Nigeria | Not specified | Not reported | Blood |
| Ndow et al., 2023 [82] | Cross sectional; Prospective | Hospital-based | Gambia | 2020-2022 | Lateral flow chromatographic immunoassay | Blood |
| Ndububa et al., 2022 [83] | Cohort ; Prospective | Hospital-based | Nigeria | Nov/2015-Mar/2021 | Lateral flow chromatographic immunoassay | Blood |
| Njoku et al., 2020 [84] | Cross sectional; Prospective | Hospital-based | Nigeria | Mar/2016-Jun/2016 | ELISA | Blood |
| Njoku et al., 2015 [85] | Cross sectional; Prospective | Hospital-based | Nigeria | Not specified | ELISA | Blood |
| Nkrumah et al., 2011 [86] | Cross sectional; Prospective | Hospital-based | Ghana | Not specified | Lateral flow chromatographic immunoassay | Blood |
| Nongo et al., 2016 [87] | Cross sectional; Prospective | Hospital-based | Nigeria | Not specified | ELISA | Blood |
| Nwuzo et al., 2020 [88] | Cross sectional; Prospective | Hospital-based | Nigeria | Not specified | ELISA | Blood |
| Obi et al., 1993 [89] | Cross sectional; Prospective | Hospital-based | Nigeria | Not specified | Lateral flow chromatographic immunoassay | Blood |
| Obi et al., 2012 [90] | Cross sectional; Prospective | Hospital-based | Nigeria | Mar/2010-Aug/2010 | Rapid diagnostic tests | Blood |
| Obi et al., 2006 [91] | Cross sectional; Prospective | Hospital-based | Nigeria | Jan/2000-Dec/2004 | Rapid diagnostic tests | Blood |
| Obi et al., 2006 [92] | Case control; Prospective | Hospital-based | Nigeria | Jan/2005-Aug/2005 | Not reported | Blood |
| Odjiegbe et al., 2015 [93] | Cross sectional; Prospective | Hospital-based | Nigeria | Not specified | Immunochromatographic test | Blood |
| Okafor et al., 1979 [94] | Cross sectional; Prospective | Hospital-based | Nigeria | Not specified | Agglutination test | Blood |
| Okeke et al., 2012 [95] | Cross sectional; Retrospective | Hospital-based | Nigeria | Jan/2007-Dec/2009 | ELISA | Blood |
| Olakunde et al., 2021 [96] | Cross sectional; Prospective | Hospital-based | Nigeria | 2019 | Not reported | Blood |
| Olaleye et al., 2015 [97] | Cross sectional; Prospective | Hospital-based | Nigeria | Sep/2011-Dec/2011 | Lateral flow chromatographic immunoassay | Blood |
| Olaolu et al., 2023 [98] | Cross sectional; Prospective | Hospital-based | Nigeria | Apr/2021-Jun/2021 | ELISA | Blood |
| Olofinsae et al., 2014 [99] | Cross sectional; Prospective | Hospital-based | Nigeria | Not specified | ELISA | Blood |
| Olokoba et al., 2011 [100] | Cross sectional; Prospective | Hospital-based | Nigeria | Jul/2008-Dec/2008 | ELISA | Blood |
| Oluremi et al., 2020 [101] | Cross sectional; Prospective | Hospital-based | Nigeria | Feb/2019-Dec/2019 | ELISA | Blood |
| Omalu et al., 2012 [102] | Cross sectional; Prospective | Hospital-based | Nigeria | Jul/2011-Nov/2011 | ELISA | Blood |
| Omatola et al., 2021 [103] | Cross sectional; Prospective | Hospital-based | Nigeria | Sep/2019-Nov/2019 | Rapid diagnostic tests | Blood |
| Omatola et al., 2019 [104] | Cross sectional; Prospective | Hospital-based | Nigeria | Sep/2017-Nov/2017 | Lateral flow chromatographic immunoassay | Blood |
| Omote et al., 2020 [105] | Cross sectional; Prospective | Hospital-based | Nigeria | May/2019-Aug/2019 | Lateral flow chromatographic immunoassay | Blood |
| Onakewhor et al., 2001 [106] | Cross sectional; Prospective | Hospital-based | Nigeria | Nov/1997-Apr/1998 | ELISA | Blood |
| Onzulike et al., 2007 [107] | Cross sectional; Prospective | Hospital-based | Nigeria | Sep/2006-Nov/2006 | ELISA | Blood |
| Onwere et al., 2012 [108] | Cross sectional; Prospective | Hospital-based | Nigeria | Jun/2010-Nov/2010 | ELISA | Blood |
| Opaleye et al., 2016 [109] | Cross sectional; Prospective | Hospital-based | Nigeria | Not specified | ELISA | Blood |
| Ouermi et al., 2009 [110] | Cross sectional; Prospective | Hospital-based | Burkina Faso | Jan/2009-Jun/2009 | ELISA | Blood |
| Ouoba et al., 2023 [111] | Cross sectional; Prospective | Hospital-based | Burkina Faso | Feb/2021- Nov/2021 | Chemiluminescence assay | Blood |
| Ouoba et al., 2022 [112] | Cross sectional; Prospective | Hospital-based | Burkina Faso | Feb/2021-Nov/2021 | Chemiluminescence assay | Blood |
| Pennap et al., 2015 [113] | Cross sectional; Prospective | Hospital-based | Nigeria | May/2014-Dec/2014 | Rapid diagnostic tests | Blood |
| Pennap et al., 2011 [114] | Cross sectional; Prospective | Hospital-based | Nigeria | Avr/2009-Sep/2009 | ELISA | Blood |
| Rabiu et al., 2010 [115] | Case control; Prospective | Hospital-based | Nigeria | Aug/2006-Jan/2007 | Not reported | Blood |
| Roingeard et al., 1993 [116] | Cross sectional; Prospective | Hospital-based | Senegal | Not specified | ELISA | Blood |
| Rouet et al., 2004 [117] | Cross sectional; Prospective | Hospital-based | Ivory Coast | Sep/1995-Nov/1998 | ELISA | Blood |
| Sangare et al., 2009 [118] | Cross sectional; Prospective | Hospital-based | Burkina Faso | Jul/2005-Aug/2005 | ELISA | Blood |
| Sangare et al., 2011 [119] | Cross sectional; Prospective | Hospital-based | Burkina Faso | Oct/2006-Jan/2007 | ELISA | Blood |
| Sanou et al., 2018 [120] | Cross sectional; Prospective | Hospital-based | Burkina Faso | Oct/2013-Dec/2014 | ELISA | Blood |
| Shuaibu et al., 2021 [121] | Cross sectional; Prospective | Hospital-based | Nigeria | Aug/2017-Jan/2018 | ELISA | Blood |
| Sidibe et al., 2001 [122] | Cross sectional; Prospective | Hospital-based | Mali | Not specified | Radioimmunoassay | Blood |
| Simpore et al., 2006 [123] | Cross sectional; Prospective | Hospital-based | Burkina Faso | Apr/2003-May/2005 | ELISA | Blood |
| Simpore et al., 2006 [124] | Cross sectional; Prospective | Hospital-based | Burkina Faso | May/2004-Aug/2005 | Immunoassay kit | Blood |
| Talla et al., 2021 [125] | Cross sectional; Prospective | Hospital-based | Nigeria | Jun/2016-Oct/2018 | Rapid diagnostic tests | Blood |
| Torlesse et al., 1997 [126] | Cross sectional; Prospective | Hospital-based | Sierra Leone | Not specified | Immunochromatographic test | Blood |
| Ugbebor et al., 2011 [127] | Cross sectional; Prospective | Hospital-based | Nigeria | Oct/2009- Oct/2010 | ELISA | Blood |
| Ugwu et al., 2022 [128] | Cross sectional; Prospective | Hospital-based | Nigeria | May/2020-Apr/2021 | ELISA | Blood |
| Ukwu et al., 2015 [129] | Cross sectional; Prospective | Hospital-based | Nigeria | Dec/2012-Oct/2013 | ELISA | Blood |
| Umoke et al., 2021 [130] | Cross sectional; Prospective | Hospital-based | Nigeria | Jan/2018-Dec/2018 | ELISA | Blood |
| Usanga et al., 2011 [131] | Cross sectional; Prospective | Hospital-based | Nigeria | 2007-2008 | Rapid diagnostic tests | Blood |
| Utoo et al., 2013 [132] | Cross sectional; Prospective | Hospital-based | Nigeria | Jan/2010-Jun/2010 | ELISA | Blood |
| Volker et al., 2017 [133] | Cross sectional; Prospective | Hospital-based | Ghana | Oct/2011-Jan/2012 | ELISA | Blood |
| Wurie et al., 2005 [134] | Cross sectional; Prospective | Hospital-based | Sierra Leone | Not specified | Agglutination test | Blood |
| Yakasai et al., 2012 [135] | Case control; Prospective | Hospital-based | Nigeria | Jan/2011-Apr/2011 | ELISA | Blood |
| Yelemkoure et al., 2018 [136] | Cross sectional; Prospective | Hospital-based | Burkina Faso | Sep/2013-Jun/2014 | PCR | Blood |
| Yendewa et al., 2021 [137] | Cross sectional; Prospective | Hospital-based | Sierra Leone | Feb/2019-May/2019 | Immunoassay kit | Blood |
| Yoon et al., 2012 [138] | Cross sectional; Prospective | Hospital-based | Ghana | Dec/2008-Mar/2009 | Not reported | Blood |

References

1. Aba, H.O., C.M.Z. Whong, and M. Aminu, *Co-infection of human immunodeficiency virus and hepatitis B surface antigen among pregnant women in Kaduna, Nigeria.* Immunology, 2012. **137**(SUPPL. 1): p. 514-515.

2. Aba, H.O. and M. Aminu, *Seroprevalence of hepatitis B virus serological markers among pregnant Nigerian women.* Annals of African medicine, 2016. **15**(1): p. 20-7.

3. Abdullahi, B. and M. Bilkisu, *Seroprevalence of hepatitis B infection among pregnant women in Katsina, Nigeria.* International Journal of Natural and Applied Sciences, 2011. **7**(4): p. 425-428.

4. Aboubakar, M., et al., *Prevention and factors associated with anti-HCV carriage in pregnant women living in Cotonou.* Pan African Medical Journal, 2020. **36**.

5. Abuku, V.G., E.A. Allotey, and M. Akonde, *Clinical and laboratory presentation of first-time antenatal care visits of pregnant women in Ghana, a hospital-based study.* PloS one, 2023. **18**(1): p. e0280031.

6. Abulude, O.A., I.l. Ahmed, and F.U. Sadisu, *Assessment of Hepatitis B Viral Infection as a Predictor of Hepatic Enzymes and Compounds Alteration among Antenatal Patients.* Medical sciences (Basel, Switzerland), 2017. **5**(4).

7. Acquaye, J.K. and J.A. Mingle, *Hepatitis B viral markers in Ghanaian pregnant women.* West African journal of medicine, 1994. **13**(3): p. 134-7.

8. Adegbesan-Omilabu, M.A., et al., *Seroprevalence of hepatitis B virus infection among pregnant women at the antenatal booking clinic of a Tertiary Hospital in Lagos Nigeria.* Nigerian journal of clinical practice, 2015. **18**(6): p. 819-23.

9. Adeogun, O.S., et al., *Incidence of HIV, hepatitis B and C, and their co-infections among pregnant women attending selected general hospitals in Ondo State.* Acta Microbiologica Bulgarica, 2020. **36**(2): p. 53-58.

10. Adesina, O., et al., *Human immuno-deficiency virus and hepatitis B virus coinfection in pregnancy at the University College Hospital, Ibadan.* African journal of medicine and medical sciences, 2010. **39**(4): p. 305-10.

11. Adeyemi, A.B., et al., *Prevalence of antenatal hepatitis B infection in tertiary and non-tertiary health facilities in Ibadan, Nigeria.* Nigerian journal of medicine : journal of the National Association of Resident Doctors of Nigeria, 2014. **23**(3): p. 248-53.

12. Adjei, C.A., et al., *Hepatitis B Infection among Parturient Women in Peri-Urban Ghana.* The American journal of tropical medicine and hygiene, 2018. **99**(6): p. 1469-1474.

13. Adu-Sarkodie, Y., et al., *Seroprevalence of hepatitis B markers in a rural community in Ghana.* Tropical Medicine, 1996. **38**(3-4): p. 87-90.

14. Agbozo, F., A. Abubakari, and A. Jahn, *Maternal morbidities in Ghana: Risk factors and effect on newborn health outcomes.* International Journal of Gynecology and Obstetrics, 2018. **143**(Supplement 3): p. 190.

15. Aigere, E.O.S., et al., *Clinico-epidemiological correlates of Hepatitis B infection in suburban population of pregnant women in Niger Delta region of Nigeria.* Nigerian Quarterly Journal of Hospital Medicine, 2013. **23**(3): p. 205-209.

16. Ajayi, B.B., et al., *Seroprevalence of some sexually transmitted infections among antenatal attendees in university of Maiduguri teaching hospital, Maiduguri-Nigeria.* Annals of Biological Research, 2013. **4**(2): p. 141-145.

17. Ajileye, A.B., et al., *Seroprevalence of HIV, HBsAg, HCV and VDRL among pregnant women in Abule-Egba, Lagos state, Nigeria.* Journal of Experimental Research, 2020. **8**(4): p. 45-51.

18. Akani, C.I., et al., *Sero-prevalence of hepatitis B surface antigen (HBsAg) in pregnant women in Port Harcourt, Nigeria.* The Nigerian postgraduate medical journal, 2005. **12**(4): p. 266-70.

19. Alassan, K.S., et al., *[Seroprevalence and factors associated with viral hepatitis B among pregnant women in Parakou, Republic of Benin].* Seroprevalence et facteurs associes a l'hepatite virale B chez les gestantes a Parakou en Republique du Benin., 2019. **33**: p. 226.

20. Aluor, E.P.T., et al., *SERO-epidemiological survey and risk factors for Hepatitis B virus (HBV) infection among pregnant women in Logo LGA, Benue State, Nigeria.* African Journal of Clinical and Experimental Microbiology, 2016. **17**(1): p. 66-75.

21. Anabire, N.G., O. Quaye, and G.K. Helegbe, *Circulation of multiple hepatitis B virus genotypes in individual pregnant women seeking antenatal care in northern Ghana.* Virology journal, 2023. **20**(1): p. 149.

22. Anaedobe, C.G., et al., *Prevalence, socio-demographic features and risk factors of Hepatitis B virus infection among pregnant women in Southwestern Nigeria.* Pan African Medical Journal, 2015. **20**: p. 406.

23. Andernach, I.E., et al., *Characterization of hepatitis delta virus in sub-Saharan Africa.* Journal of clinical microbiology, 2014. **52**(5): p. 1629-36.

24. Antuamwine, B.B., E.D. Herchel, and E.M. Bawa, *Comparative prevalence of hepatitis B virus infection among pregnant women accessing free maternal care in a tertiary hospital in Ghana.* PloS one, 2022. **17**(3): p. e0263651.

25. Apea-Kubi, K.A., et al., *HTLV-1 and other viral sexually transmitted infections in antenatal and gynaecological patients in Ghana.* West African journal of medicine, 2006. **25**(1): p. 17-21.

26. Atilola, G., et al., *Epidemiology of HBV in Pregnant Women, South West Nigeria.* Journal of epidemiology and global health, 2018. **8**(3-4): p. 115-123.

27. Ayoola, E.A., O. Ogunbode, and H.A. Odelola, *Congenital transmission of hepatitis B antigen in Nigerians.* Archives of virology, 1981. **67**(1): p. 97-9.

28. Baba, M.M., I.S. Onwuka, and S.S. Baba, *Hepatitis B and C virus infections among pregnant women in Maiduguri, Nigeria.* Central European Journal of Public Health, 1999. **7**(2): p. 60-62.

29. Bejide, I.O., et al., *Seroprevalence of Hepatitis B virus and human immunodeficiency virus co-infection in pregnant women from Osun State, Nigeria.* Journal of infection in developing countries, 2024. **18**(1): p. 145-151.

30. Bigot, K.A., N. Kodjoh, and I.S. Zohoun, *Seroprevalence of the surface antigen (HBsAg) of hepatitis B virus in pregnant women and their children.* Seroprevalence de l'antigene HBs du virus de l'hepatite B chez les femmes enceintes et leurs enfants., 1992. **39**(7): p. 487-490.

31. Bittaye, M., et al., *Hepatitis B virus sero-prevalence amongst pregnant women in the Gambia.* BMC infectious diseases, 2019. **19**(1): p. 259.

32. Buseri, F., E. Seiyaboh, and Z. Jeremiah, *Surveying Infections among Pregnant Women in the Niger Delta, Nigeria.* Journal of global infectious diseases, 2010. **2**(3): p. 203-11.

33. Candotti, D., K. Danso, and J.-P. Allain, *Maternofetal transmission of hepatitis B virus genotype E in Ghana, west Africa.* The Journal of general virology, 2007. **88**(Pt 10): p. 2686-2695.

34. Cho, Y., et al., *The prevalence and risk factors for hepatitis B surface ag positivity in pregnant women in eastern region of ghana.* Gut and liver, 2012. **6**(2): p. 235-40.

35. Collenberg, E., et al., *Seroprevalence of six different viruses among pregnant women and blood donors in rural and urban Burkina Faso: A comparative analysis.* Journal of medical virology, 2006. **78**(5): p. 683-92.

36. Damale, N.K.R., A.T. Lassey, and V. Bekoe, *Hepatitis B virus seroprevalence among parturients in Accra, Ghana.* International journal of gynaecology and obstetrics: the official organ of the International Federation of Gynaecology and Obstetrics, 2005. **90**(3): p. 240-1.

37. Dao, B., et al., *[HIV infection and hepatitis B co-infection: survey of prevalence in pregnant women in Bobo Dioulasso, Burkina Faso].* Co-infection hepatite B et VIH: enquete de preevalence chez les femmes enceintes a Bobo Dioulasso, Burkina Faso., 2001. **22**(2): p. 83-6.

38. De Paschale, M., et al., *Prevalence of HBV, HDV, HCV, and HIV infection during pregnancy in northern Benin.* Journal of medical virology, 2014. **86**(8): p. 1281-7.

39. Dortey, B.A., et al., *Seroprevalence of Hepatitis B virus infection and associated factors among pregnant women at Korle-Bu Teaching Hospital, Ghana.* PloS one, 2020. **15**(4): p. e0232208.

40. Doumbia, K., et al., *[Infection with the Hepatitis B virus in pregnant women in the Gynecology and Obstetrics Department of the Gabriel Toure University Hospital Center].* L'Infection par le virus de L'Hepatite B chez la femme enceinte dans le service de Gynecologie-Obstetrique du Centre Hospitalo-Universitaire Gabriel Toure., 2022. **37**(2): p. 56-60.

41. Eduku, A. and V.E. Senoo-Dogbey, *Seroprevalence of hepatitis B virus infection (HBsAg) and associated factors among antenatal clinic attendees in a secondary-level facility in southern Ghana.* Clinical Epidemiology and Global Health, 2024. **26**: p. 101553.

42. Eke, A.C., et al., *Prevalence, correlates and pattern of hepatitis B surface antigen in a low resource setting.* Virology journal, 2011. **8**: p. 12.

43. Ephraim, R., et al., *Seroprevalence and risk factors of Hepatitis B and Hepatitis C infections among pregnant women in the Asante Akim North Municipality of the Ashanti region, Ghana; a cross sectional study.* African health sciences, 2015. **15**(3): p. 709-13.

44. Erhabor, O., et al., *Prevalence of some hepatitis B virus markers among pregnant women attending antenatal clinic in Specialist Hospital Sokoto Nigeria.* Human antibodies, 2020. **28**(3): p. 233-243.

45. Evelyn, M.E., et al., *Effects of hepatitis B infection on haemtological parameters in pregnancy in Port Harcourt, Nigeria.* Research Journal of Medical Sciences, 2009. **3**(6): p. 194-197.

46. Ezechi, O.C., et al., *Sero-prevalence and factors associated with Hepatitis B and C co-infection in pregnant Nigerian women living with HIV infection.* The Pan African medical journal, 2014. **17**: p. 197.

47. Faleye, T.O.C., et al., *Molecular epidemiology of hepatitis B virus among pregnant women in southwestern Nigeria.* International Journal of Infectious Diseases, 2014. **21**(SUPPL. 1): p. 332.

48. Faleye, T.O.C., et al., *Detection of hepatitis B virus isolates with mutations associated with immune escape mutants among pregnant women in Ibadan, southwestern Nigeria.* SpringerPlus, 2015. **4**: p. 43.

49. Fofana, D.B., et al., *PREVALENCE AND EVALUATION OF HEPATITIS B VIRAL REPLICATION IN PREGNANT WOMEN IN MALI.* Topics in Antiviral Medicine, 2023. **31**(2): p. 237.

50. Fowotade, A., et al., *Hepatitis B virus infection among pregnant women on antenatal visits: Rapid tests or ELISA?* African Journal of Clinical and Experimental Microbiology, 2021. **22**(3): p. 352-358.

51. Frempong, M.T., et al., *Hepatitis B and C infections in HIV-1 and non-HIV infected pregnant women in the Brong-Ahafo Region, Ghana.* PloS one, 2019. **14**(7): p. e0219922.

52. Ghazzawi, M., et al., *Factors Associated with HBsAg Seropositivity among Pregnant Women Receiving Antenatal Care at 10 Community Health Centers in Freetown, Sierra Leone: A Cross-Sectional Study.* Pathogens (Basel, Switzerland), 2022. **11**(2).

53. Guingane, A.N., et al., *Screening for Hepatitis B in partners and children of women positive for surface antigen, Burkina Faso.* Bulletin of the World Health Organization, 2022. **100**(4): p. 256-267.

54. Harry, T.O., M.D. Bajani, and A.E. Moses, *Hepatitis B virus infection among blood donors and pregnant women in Maiduguri, Nigeria.* East African medical journal, 1994. **71**(9): p. 596-7.

55. Helegbe, G.K., et al., *Seroprevalence of Malaria and Hepatitis B Coinfection among Pregnant Women in Tamale Metropolis of Ghana: A Cross-Sectional Study.* The Canadian journal of infectious diseases & medical microbiology = Journal canadien des maladies infectieuses et de la microbiologie medicale, 2018. **2018**: p. 5610981.

56. Ifeorah, I.M., et al., *Patterns of serologic markers of hepatitis B virus infection and the risk of transmission among pregnant women in southwestern Nigeria.* Journal of immunoassay & immunochemistry, 2017. **38**(6): p. 639-651.

57. Ikeme, A.C., H.U. Ezegwui, and C. Ogbonna, *Sero prevalence of hepatitis B surface antigen (HBsAg) in pregnant women in Southeast Nigeria.* Tropical doctor, 2006. **36**(2): p. 128.

58. Iklaki, C.U., et al., *Sero-prevalence of hepatitis B infection and its risk factors among women admitted for delivery in Ucth, Calabar, Nigeria.* British Journal of Medicine and Medical Research, 2015. **8**(4): p. 324-333.

59. Ilboudo, D., A. Sawadogo, and J. Simpore, *Mother-child transmission of hepatitis B virus in Ouagadougou, Burkina Faso.* Transmission mere-enfant du virus de l'hepatite B, a Ouagadougou, Burkina Faso., 2002. **62**(1): p. 99-100.

60. Ilboudo, D., et al., *Prevalence of human herpes virus-8 and hepatitis B virus among HIV seropositive pregnant women enrolled in the Mother-to-Child HIV Transmission Prevention Program at Saint Camille Medical Centre in Burkina Faso.* Pakistan journal of biological sciences : PJBS, 2007. **10**(17): p. 2831-7.

61. Ilboudo, D., et al., *Towards the complete eradication of mother-to-child HIV/HBV coinfection at Saint Camille Medical Centre in Burkina Faso, Africa.* The Brazilian journal of infectious diseases : an official publication of the Brazilian Society of Infectious Diseases, 2010. **14**(3): p. 219-24.

62. Jatau, E.D. and A. Yabaya, *Sero prevalence of Hepatitis B virus in pregnant women attending a clinic in Zaria, Nigeria.* Science World Journal, 2009. **4**(2): p. 7-9.

63. Kolawole, O.M., et al., *Seroprevalence of hepatitis B surface antigenemia and its effects on hematological parameters in pregnant women in Osogbo, Nigeria.* Virology journal, 2012. **9**: p. 317.

64. Kouakou, C., et al., *[Mother-to-child hepatitis B virus markers transmission at a reference hospital in Cote d'Ivoire].* Transmission mere - enfant des marqueurs du virus de l'hepatite B dans un hopital de reference en Cote d'Ivoire., 2020. **35**(2): p. 43-46.

65. Kuugbee, E.D., et al., *Seroprevalence and Risk Factors of Sexually Transmitted Blood-Borne Infections among Pregnant Women Attending Antenatal Care in Jirapa, Upper West Region of Ghana.* The Canadian journal of infectious diseases & medical microbiology = Journal canadien des maladies infectieuses et de la microbiologie medicale, 2023. **2023**: p. 3157202.

66. Kwadzokpui, P.K., et al., *Prevalence and Knowledge of Hepatitis B Virus Infection among Pregnant Women in the Ningo-Prampram District, Ghana.* International journal of hepatology, 2020. **2020**: p. 7965146.

67. Lar, P.M., et al., *Prevalence and immune status of HIV/HBV co-infected pregnant women.* African Journal of Clinical and Experimental Microbiology, 2013. **14**(3): p. 120-126.

68. Lo, G., et al., *Prevalence of surface antigen of hepatitis B (HBsAg) in pregnant women in Hospital Laboratory Military Ouakam (HMO), Dakar.* Prevalence de l'antigene de surface du virus de l'hepatite B (AgHBs) chez les femmes enceintes au laboratoire de l'hopital Militaire de Ouakam (HMO), Dakar., 2012. **59**(5): p. 241-244.

69. Lohoues, K., et al., *Transmission in utero of the hepatitis B virus in ivory coast the case for mass vaccination.* Sante (Montrouge, France), 1998. **8**(6): p. 401-4.

70. Luuse, A., et al., *Sero-Prevalence of Hepatitis B Surface Antigen Amongst Pregnant Women Attending an Antenatal Clinic, Volta Region, Ghana.* Journal of public health in Africa, 2016. **7**(2): p. 584.

71. MacLean, B., et al., *Seroprevalence of hepatitis B surface antigen among pregnant women attending the hospital for women & children in Koutiala, Mali.* South African Medical Journal, 2012. **102**(1): p. 47-49.

72. Magaji, F.A., et al., *Prevalence of hepatitis B virus infection in pregnant women with and without HIV in Jos, Nigeria.* International journal of infectious diseases : IJID : official publication of the International Society for Infectious Diseases, 2021. **104**: p. 276-281.

73. Maiga, Y.I., et al., *[Transmission of hepatitis B virus from mother to child in Bamako-Mali].* Transmission du virus B de l'hepatite de la mere a l'enfant a Bamako au Mali., 1992. **85**(1): p. 5-9.

74. Mamadou, S., et al., *HIV infection and hepatitis B seroprevalence among antenatal clinic attendees in Niger, West Africa.* HIV/AIDS (Auckland, N.Z.), 2012. **4**: p. 1-4.

75. Mansour, W., et al., *Prevalence, risk factors, and molecular epidemiology of hepatitis B and hepatitis delta virus in pregnant women and in patients in Mauritania.* Journal of medical virology, 2012. **84**(8): p. 1186-98.

76. Marinier, E., et al., *Lack of perinatal transmission of hepatitis B virus infection in Senegal, West Africa.* The Journal of pediatrics, 1985. **106**(5): p. 843-9.

77. Mbaawuaga, E.M., et al., *Hepatitis B Virus (HBV) infection among pregnant women in Makurdi, Nigeria.* African Journal Biomedical Research, 2008. **11**(2): p. 155-159.

78. Mustapha, G.U., et al., *Seroprevalence of hepatitis B virus among antenatal clinic attendees in Gamawa Local Government Area, Bauchi State, Nigeria.* BMC infectious diseases, 2020. **20**(1): p. 194.

79. Nacro, B., et al., *[HBs antigen carrier state in pregnant women in Bobo Dioulasso (Burkina Faso)].* Portage de l'antigene HBs chez les femmes enceintes a Bobo-Dioulasso (Burkina Faso). 2000. **45**(2): p. 188-90.

80. Ndako, J.A., et al., *Hepatitis B virus sero-prevalence among pregnant females in northern Nigeria.* Research Journal of Medical Sciences, 2012. **6**(3): p. 129-133.

81. Ndams, I.S., et al., *Biochemical analysis of HIV and HBV infected pregnant women in Minna, Nigeria.* Journal of Pure and Applied Microbiology, 2009. **3**(2): p. 473-476.

82. Ndow, G., et al., *Estimating the residual risk of hepatitis B mother-to-child transmission in The Gambia, 30 years after HBV vaccine implementation.* Journal of Hepatology, 2023. **78**(Supplement 1): p. S72-S73.

83. Ndububa, D., et al., *Prospective cohort study of prevention of mother to child transmission of hepatitis B infection and 9 months follow-up of hepatitis B-exposed infants at Ile-Ife, Nigeria.* BMJ open, 2022. **12**(11): p. e063482.

84. Njoku, C., et al., *Human Immunodeficiency and Hepatitis B Viral Co-infection in Women Attending Antenatal Care Clinic in a Tertiary Health Institution in Nigeria.* International Journal of Medical Research & Health Sciences, 2020. **9**(3): p. 8-17.

85. Njoku, C.O., et al., *Screening rate, prevalence and complications of hepatitis B virus infection among women at delivery in UCTH Calabar, Nigeria.* Scholars Journal of Applied Medical Sciences, 2015. **3**(3E): p. 1404-1410.

86. Nkrumah, C. and W.K.B.A. Owiredu, *Prevalence of hepatitis B surface antigen (HBsAg) in pregnant women attending antenatal clinic (ANC) in Wenchi.* Clinical Chemistry, 2011. **57**(10 SUPPL. 1): p. A124.

87. Nongo, B.H., et al., *Seroprevalence of hepatitis B virus among antenatal attendees at the University of Abuja Teaching Hospital, Nigeria.* Annals of Nigerian Medicine, 2016. **10**(2): p. 58-62.

88. Nwuzo, A.C., et al., *Prevalence of hepatitis B virus and HIV infections among pregnant women visiting healthcare institutions in Ebonyi State, Nigeria.* Scientific Research and Essays, 2020. **15**(2): p. 18-25.

89. Obi, C.L., et al., *A comparison of human immunodeficiency virus (HIV) seropositivity and hepatitis B surface antigenemia (HBs Ag) among the same group of apparently healthy pregnant women in Lagos, Nigeria: a preliminary report.* Viral immunology, 1993. **6**(1): p. 43-7.

90. Obi, R.K., et al., *A prospective study of three blood-borne viral pathogens among pregnant women attending ante-natal care in Owerri, Nigeria.* Journal of Public Health and Epidemiology, 2012. **4**(9): p. 226-229.

91. Obi, R.K., et al., *Prevalence of hepatitis B virus infection among pregnant women in an antenatal clinic in Port Harcourt, Nigeria.* African Journal of Clinical and Experimental Microbiology, 2006. **7**(2): p. 78-82.

92. Obi, S.N., H.E. Onah, and F.O. Ezugwu, *Risk factors for hepatitis B infection during pregnancy in a Nigerian obstetric population.* Journal of obstetrics and gynaecology : the journal of the Institute of Obstetrics and Gynaecology, 2006. **26**(8): p. 770-2.

93. Ojiegbe, N., et al., *Seroprevalence of hepatitis b virus infection and infectivity status among pregnant women in Umuahia, South-East Nigeria.* International Journal of Gynecology and Obstetrics, 2015. **131**(SUPPL. 5): p. E205.

94. Okafor, G.O., G.O. Obi, and W.O. Chukwudebelu, *The incidence of hepatitis B surface antigen in Nigeria.* Transactions of the Royal Society of Tropical Medicine and Hygiene, 1979. **73**(6): p. 648-50.

95. Okeke, T.C., et al., *Coinfection with hepatitis B and C viruses among HIV positive pregnant women in Enugu south east, Nigeria.* Nigerian journal of medicine : journal of the National Association of Resident Doctors of Nigeria, 2012. **21**(1): p. 57-60.

96. Olakunde, B.O., et al., *Antenatal hepatitis B screening in Nigeria: A comparative analysis with syphilis and HIV.* International journal of STD & AIDS, 2021. **32**(14): p. 1290-1297.

97. Olaleye, A., et al., *Perinatal transmission of hepatitis b virus infection in Ile-Ife, south western Nigeria.* International Journal of Gynecology and Obstetrics, 2015. **131**(SUPPL. 5): p. E205.

98. Olaolu, O.O., et al., *Seroprevalence and associated risk factors for hepatitis B virus among pregnant women attending health facility in Osogbo, Nigeria.* Rwanda Journal of Medicine and Health Sciences, 2023. **6**(2): p. 199-207.

99. Olofinsae, S.A., B.O. Ibeh, and A.A. Olufisayo, *High prevalence of hepatitis-B surface antigen in pregnant women observed in southwest Nigeria: a potential risk for vertical HbsAg transmission.* British Journal of Medicine and Medical Research, 2014. **4**(22): p. 4018-4024.

100. Olokoba, A.B., et al., *Hepatitis B virus infection amongst pregnant women in North-eastern Nigeria- a call for action.* Nigerian journal of clinical practice, 2011. **14**(1): p. 10-3.

101. Oluremi, A.S., et al., *High Viral Hepatitis Infection among Pregnant Women Attending Antenatal Clinic in Adeoyo Maternity Teaching Hospital Ibadan (AMTHI) Oyo State, Nigeria.* Journal of immunoassay & immunochemistry, 2020. **41**(5): p. 913-923.

102. Omalu, I.C.J., et al., *Seroprevalence of malaria and hepatitis B (HBsAg) with associated risk factors among pregnant women attending antenatal clinic in General Hospital Minna, North-Central Nigeria.* Annual Review and Research in Biology, 2012. **2**(4): p. 83-88.

103. Omatola, C.A. and M.-L.O. Okolo, *Hepatitis B and Asymptomatic Malaria Infection among Pregnant Women in a Semiurban Community of North-Central Nigeria.* Journal of environmental and public health, 2021. **2021**: p. 9996885.

104. Omatola, C.A., et al., *Seroprevalence of HBV, HCV, and HIV and Associated Risk Factors Among Apparently Healthy Pregnant Women in Anyigba, Nigeria.* Viral immunology, 2019. **32**(4): p. 186-191.

105. Omote, V., et al., *Awareness, risk factors and prevalence of viral Hepatitis B and C among antenatal attendees in South-Southern Nigeria: a cross-sectional and hospital-based study.* American Journal of Public Health Research, 2020. **8**(6): p. 202-208.

106. Onakewhor, J.U., E. Offor, and F.E. Okonofua, *Maternal and neonatal seroprevalence of hepatitis B surface antigen (HBsAg) in Benin City, Nigeria.* Journal of obstetrics and gynaecology : the journal of the Institute of Obstetrics and Gynaecology, 2001. **21**(6): p. 583-6.

107. Onuzulike, N. and E.O. Ogueri, *Sero-prevalence of hepatitis B surface antigen (HBsAg) in pregnant women in Owerri, Imo State of Nigeria.* Research Journal of Biological Sciences, 2007. **2**(2): p. 178-182.

108. Onwere, S., et al., *RISK FACTORS FOR HEPATITIS B VIRUS INFECTION DURING PREGNANCY IN SOUTH EASTERN NIGERIA.* East African medical journal, 2012. **89**(3): p. 89-93.

109. Opaleye, O.O., et al., *Seroprevalence of HIV, HBV, HCV, and HTLV among Pregnant Women in Southwestern Nigeria.* Journal of immunoassay & immunochemistry, 2016. **37**(1): p. 29-42.

110. Ouermi, D., et al., *Co-infection of Toxoplasma gondii with HBV in HIV-infected and uninfected pregnant women in Burkina Faso.* Pakistan journal of biological sciences : PJBS, 2009. **12**(17): p. 1188-93.

111. Ouoba, S., et al., *Intermediate hepatitis B virus infection prevalence among 1622 pregnant women in rural Burkina Faso and implications for mother-to-child transmission.* Scientific reports, 2023. **13**(1): p. 6115.

112. Ouoba, S., et al., *SEROPREVALENCE AND GENOTYPE DISTRIBUTION OF HEPATITIS B INFECTION AMONG PREGNANT WOMEN IN RURAL BURKINA FASO.* Hepatology, 2022. **76**(Supplement 1): p. S272.

113. Pennap, G.R., F.M. Ishaq, and F. Mohammed, *Parallel and overlapping hepatitis B and C virus infection among pregnant women attending antenatal in a rural clinic in northern Nigeria.* International Journal of Current Microbiology and Applied Sciences, 2015. **4**(5): p. 16-23.

114. Pennap, G.R., *Seroprevalence of hepatitis B surface antigen among pregnant women attending antenatal clinic in Federal Medical Center Keffi, Nigeria.* Journal of Antivirals and Antiretrovirals, 2011. **2011**.

115. Rabiu, K.A., et al., *Risk factors for hepatitis B virus infection among pregnant women in Lagos, Nigeria.* Acta obstetricia et gynecologica Scandinavica, 2010. **89**(8): p. 1024-8.

116. Roingeard, P., et al., *Perinatal transmission of hepatitis B virus in Senegal, west Africa.* Viral immunology, 1993. **6**(1): p. 65-73.

117. Rouet, F., et al., *HBV and HCV prevalence and viraemia in HIV-positive and HIV-negative pregnant women in Abidjan, Cote d'Ivoire: the ANRS 1236 study.* Journal of medical virology, 2004. **74**(1): p. 34-40.

118. Sangare, L., et al., *[Antenatal transmission of hepatitis B virus in an area of HIV moderate prevalence, Burkina Faso].* Transmission antenatale du virus de l'hepatite B en zone de prevalence moderee du VIH, Ouagadougou, Burkina Faso., 2009. **102**(4): p. 226-9.

119. Sangare, L., et al., *Importance of the confirmatory assay for the detection of the HBsAg in the epidemiological studies and in the diagnosis of the viral hepatitis B.* African Journal of Clinical and Experimental Microbiology, 2011. **12**(1): p. 44-48.

120. Sanou, A.M., et al., *Prevalence of Hepatitis B virus and Hepatitis D virus Coinfection in Western Burkina Faso and molecular characterization of the detected virus strains.* International journal of infectious diseases : IJID : official publication of the International Society for Infectious Diseases, 2018. **70**: p. 15-19.

121. Shuaibu, U.Y., et al., *Seroprevalence and factors associated with risk of hepatitis B virus infection among antenatal attendees in ABUTH Zaria, Northwestern Nigeria.* Nigerian Medical Journal, 2021. **62**(6): p. 318-324.

122. Sidibe, S., B.Y. Sacko, and I. Traore, *Prevalence of hepatitis B virus serologic markers in pregnant women in Bamako, Mali.* Prevalence des marqueurs serologiques du virus de l'hepatite B chez les femmes enceintes dans le district de Bamako, Mali., 2001. **94**(4): p. 339-341.

123. Simpore, J., et al., *Prevalence of infection by HHV-8, HIV, HCV and HBV among pregnant women in Burkina Faso [2].* Journal of Clinical Virology, 2004. **31**(1): p. 78-80.

124. Simpore, J., et al., *Prevalence of HHV-8 infections associated with HIV, HBV and HCV in pregnant women in Burkina Faso.* Journal of Medical Sciences, 2006. **6**(1): p. 93-98.

125. Talla, C., et al., *Hepatitis B infection and risk factors among pregnant women and their male partners in the Baby Shower Programme in Nigeria: a cross-sectional study.* Tropical medicine & international health : TM & IH, 2021. **26**(3): p. 316-326.

126. Torlesse, H., I.M. Wurie, and M. Hodges, *The use of immunochromatography test cards in the diagnosis of hepatitis B surface antigen among pregnant women in West Africa.* British journal of biomedical science, 1997. **54**(4): p. 256-9.

127. Ugbebor, O., et al., *The prevalence of hepatitis B and C viral infections among pregnant women.* North American journal of medical sciences, 2011. **3**(5): p. 238-41.

128. Ugwu, A.O., et al., *Seroprevalence of Hepatitis B, and C Viruses and HIV Infections among Antenatal Women in a Secondary Health Facility in Lagos, Nigeria.* West African journal of medicine, 2022. **39**(10): p. 1084-1088.

129. Ukwu, A., et al., *Outcome of pregnancy amongst hepatitis B virus positive pregnant women in a tertiary hospital, Northern Nigeria.* International Journal of Gynecology and Obstetrics, 2015. **131**(SUPPL. 5): p. E507.

130. Umoke, M., et al., *Co-infection and Risk Factors Associated with STIs among Pregnant Women in Rural Health Facilities in Nigeria: A Retrospective Study.* Inquiry : a journal of medical care organization, provision and financing, 2021. **58**: p. 46958021992912.

131. Usanga, V.U., et al., *Prevalence of sexually transmitted diseases in pregnant and non-pregnant women in Calabar, cross river state, Nigeria.* Internet Journal of Gynecology and Obstetrics, 2011. **14**(2).

132. Utoo, B.T., *Hepatitis B surface antigenemia (HBsAg) among pregnant women in southern Nigeria.* African health sciences, 2013. **13**(4): p. 1139-43.

133. Volker, F., et al., *Prevalence of pregnancy-relevant infections in a rural setting of Ghana.* BMC pregnancy and childbirth, 2017. **17**(1): p. 172.

134. Wurie, I.M., A.T. Wurie, and S.M. Gevao, *Sero-prevalence of hepatitis B virus among middle to high socio-economic antenatal population in Sierra Leone.* West African journal of medicine, 2005. **24**(1): p. 18-20.

135. Yakasai, I.A., et al., *Sero-prevalence of hepatitis B virus infection and its risk factors among pregnant women attending antenatal clinic at Aminu Kano Teaching Hospital, Kano, Nigeria.* J. basic clin. reprod. sci. (Online), 2012. **1**(1): p. 49-55.

136. Yelemkoure, E.T., et al., *Prevention of mother-to-child transmission of hepatitis B virus in Burkina Faso: Screening, vaccination and evaluation of post-vaccination antibodies against hepatitis B surface antigen in newborns.* Journal of public health in Africa, 2018. **9**(3): p. 816.

137. Yendewa, G.A., et al., *Prevalence of hepatitis B surface antigen and serological markers of other endemic infections in HIV-infected children, adolescents and pregnant women in Sierra Leone: A cross-sectional study.* International journal of infectious diseases : IJID : official publication of the International Society for Infectious Diseases, 2021. **102**: p. 45-52.

138. Yoon, H.J., et al., *Prevalence and risk factors for human immunodeficiency virus infection in pregnant women in Eastern Ghana.* Brazilian Journal of Infectious Diseases, 2012. **16**(2): p. 217-218.
