# Supplementary material for: Hepatitis B Virus Infection and Risk Factors Among Pregnant Women in Healthcare Facilities in West Africa: A Systematic Review and Meta‐Analysis
Source: Biomed Res Int. 2026 Mar 24;2026:3975525. doi: 10.1155/bmri/3975525 (PMC13140436; doi:10.1155/bmri/3975525)

S6 Table: Meta-regression analyses of prevalence of HBV in pregnant women in West Africa

| Covariate | Level | N_level | SE | Estimate | CI_low | CI_high | p_value |
| --- | --- | --- | --- | --- | --- | --- | --- |
| Year of publication | Year of publication |  | 0,007 | -0,008 | -0,022 | 0,006 | 0,240 |
| Study type | Cross sectional (Reference) | 122 |  | 0,000 |  |  |  |
|  | Case control | 3 | 0,412 | -0,286 | -1,094 | 0,522 | 0,488 |
|  | Cohort | 5 | 0,317 | 0,106 | -0,516 | 0,728 | 0,738 |
| Design | Prospective (Reference) | 127 |  | 0,000 |  |  |  |
|  | Retrospective | 3 | 0,418 | -0,414 | -1,234 | 0,405 | 0,322 |
| Countries | Nigeria (Reference) | 73 |  | 0,000 |  |  |  |
|  | Benin | 3 | 0,411 | 0,571 | -0,235 | 1,376 | 0,165 |
|  | Burkina Faso | 14 | 0,204 | 0,539 | 0,140 | 0,939 | 0,008 |
|  | Gambia | 2 | 0,493 | -0,043 | -1,009 | 0,922 | 0,930 |
|  | Ghana | 21 | 0,176 | 0,234 | -0,110 | 0,579 | 0,182 |
|  | Ivory Coast | 3 | 0,404 | 0,389 | -0,403 | 1,180 | 0,336 |
|  | Mali | 5 | 0,318 | 0,599 | -0,024 | 1,221 | 0,060 |
|  | Mauritania | 1 | 0,688 | 0,517 | -0,832 | 1,866 | 0,453 |
|  | Niger | 1 | 0,692 | 0,994 | -0,362 | 2,350 | 0,151 |
|  | Senegal | 2 | 0,488 | 0,412 | -0,545 | 1,369 | 0,399 |
|  | Sierra Leone | 4 | 0,372 | 0,437 | -0,291 | 1,166 | 0,240 |
| Vaccination period | Post-vaccination (Reference) | 49 |  | 0,000 |  |  |  |
|  | 2000-2010 | 36 | 0,145 | 0,011 | -0,272 | 0,294 | 0,940 |
|  | Pre-vaccination | 6 | 0,281 | 0,277 | -0,274 | 0,829 | 0,325 |
| HBV diagnostic method | ELISA (Reference) | 77 |  | 0,000 |  |  |  |
|  | CLIA | 1 | 0,700 | -0,321 | -1,694 | 1,051 | 0,646 |
|  | Lateral flow test | 34 | 0,151 | -0,472 | -0,769 | -0,176 | 0,002 |
|  | Latex agglutination test | 5 | 0,334 | -0,444 | -1,098 | 0,210 | 0,183 |
|  | Radioimmunoassay | 2 | 0,498 | 0,271 | -0,706 | 1,247 | 0,587 |

Buble plots of prevalence of HBV in pregnant women in West Africa


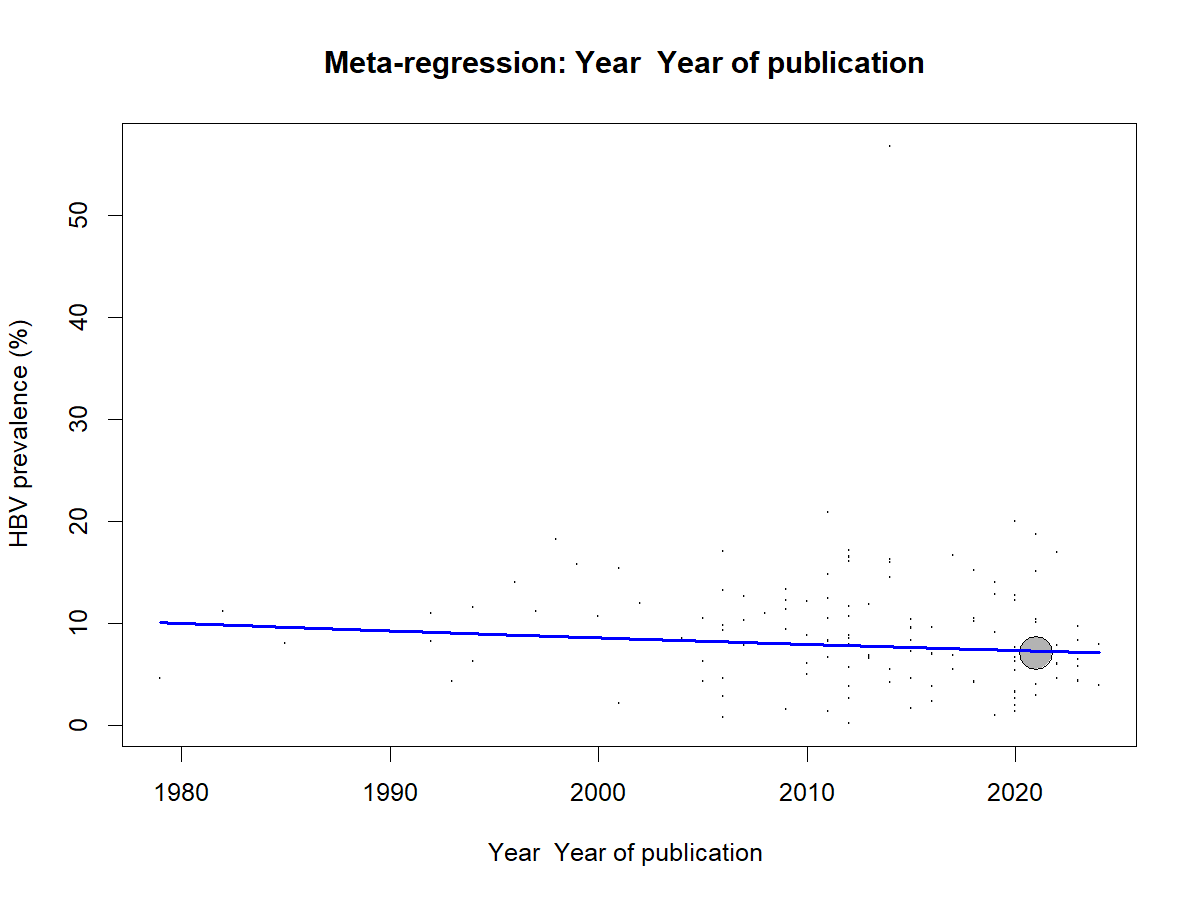


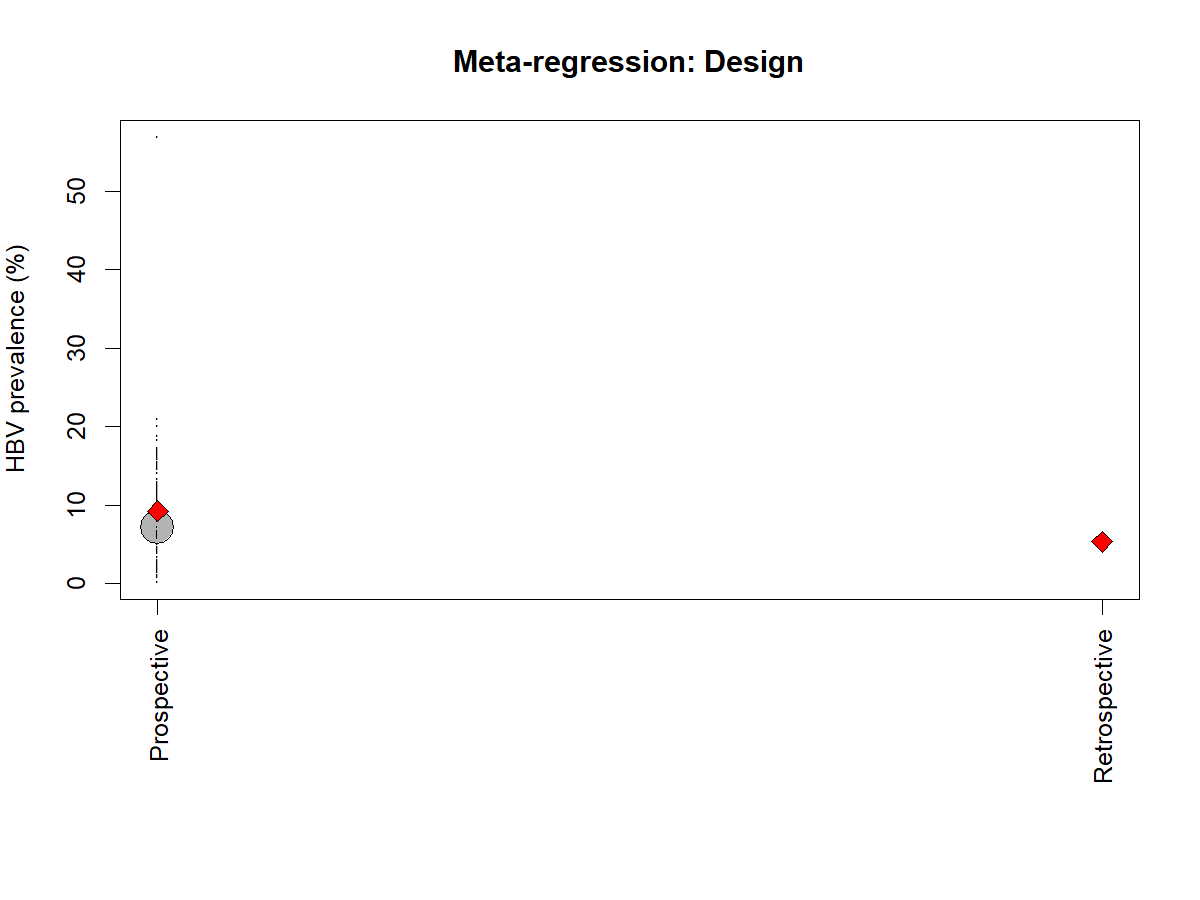


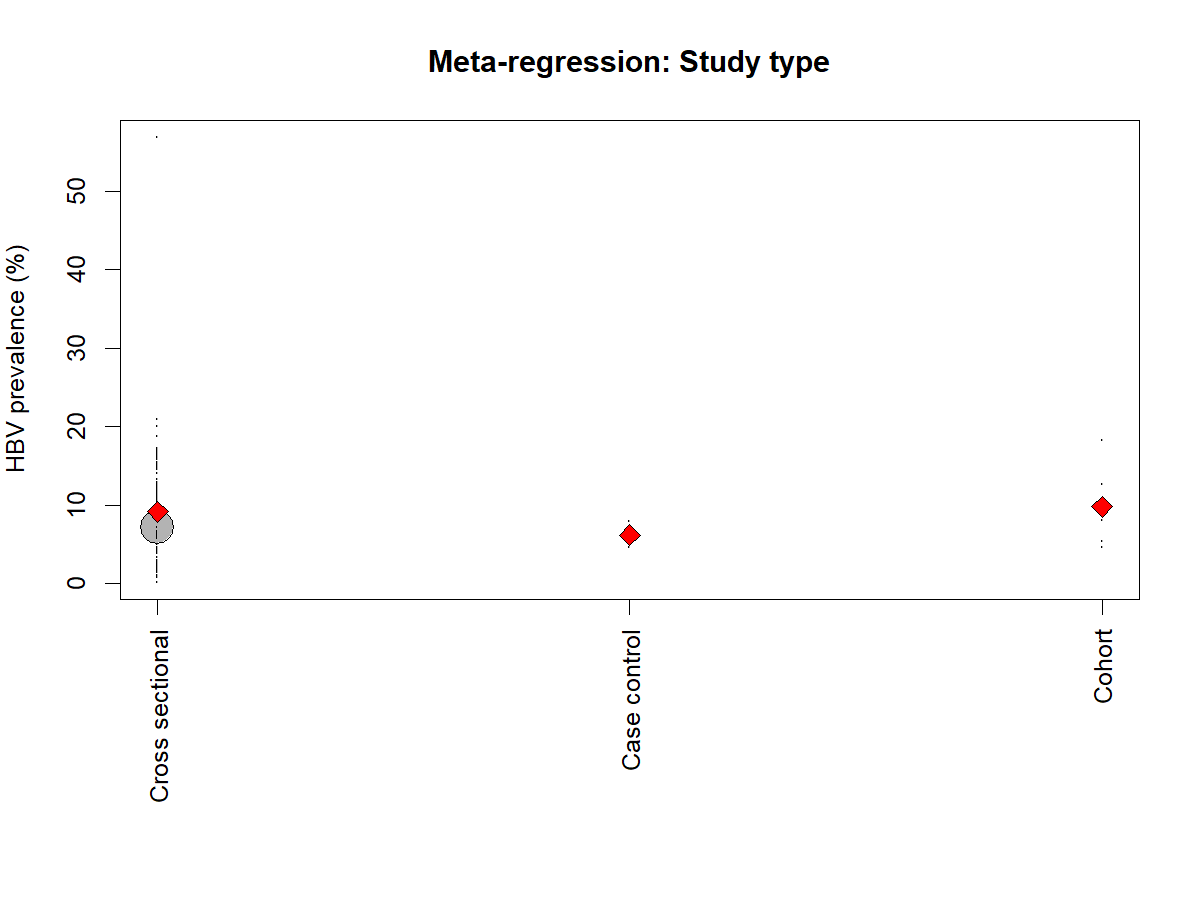


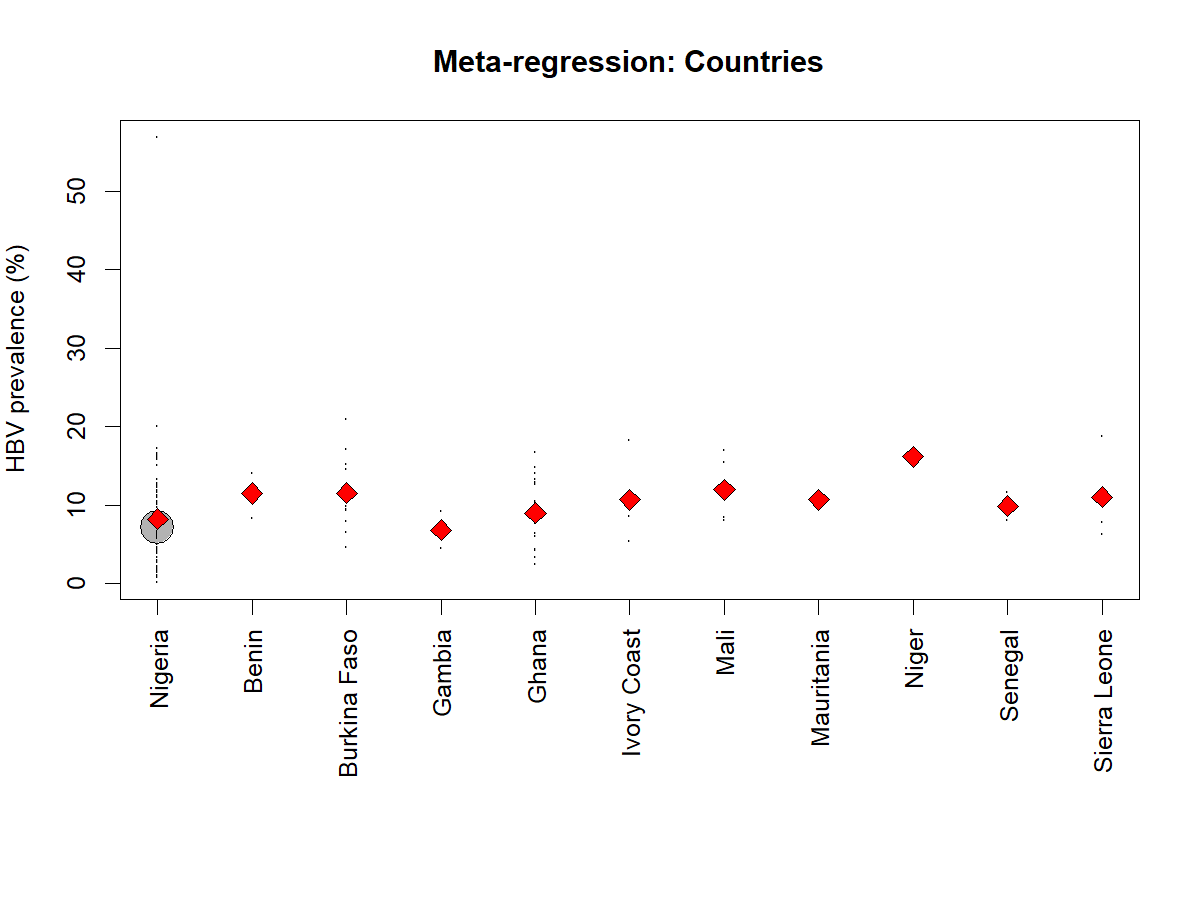


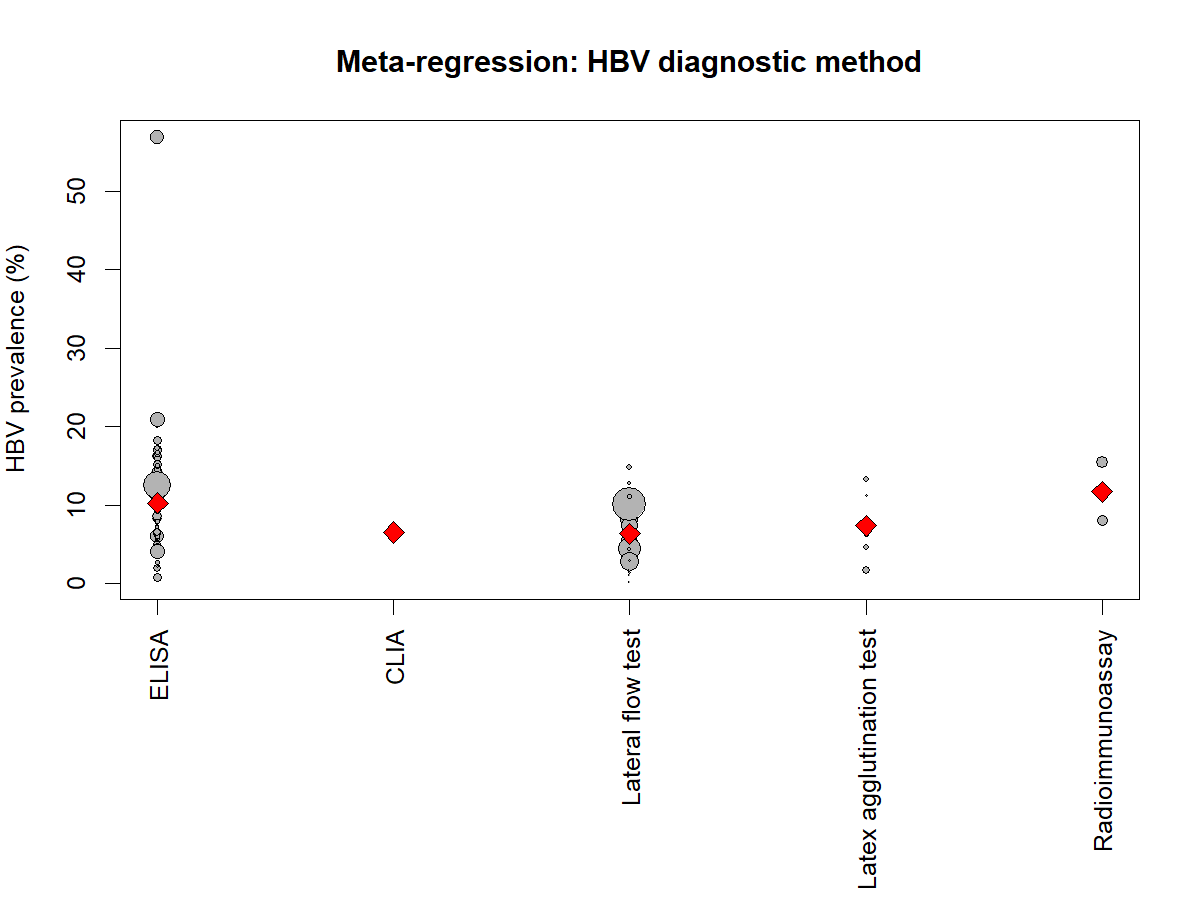


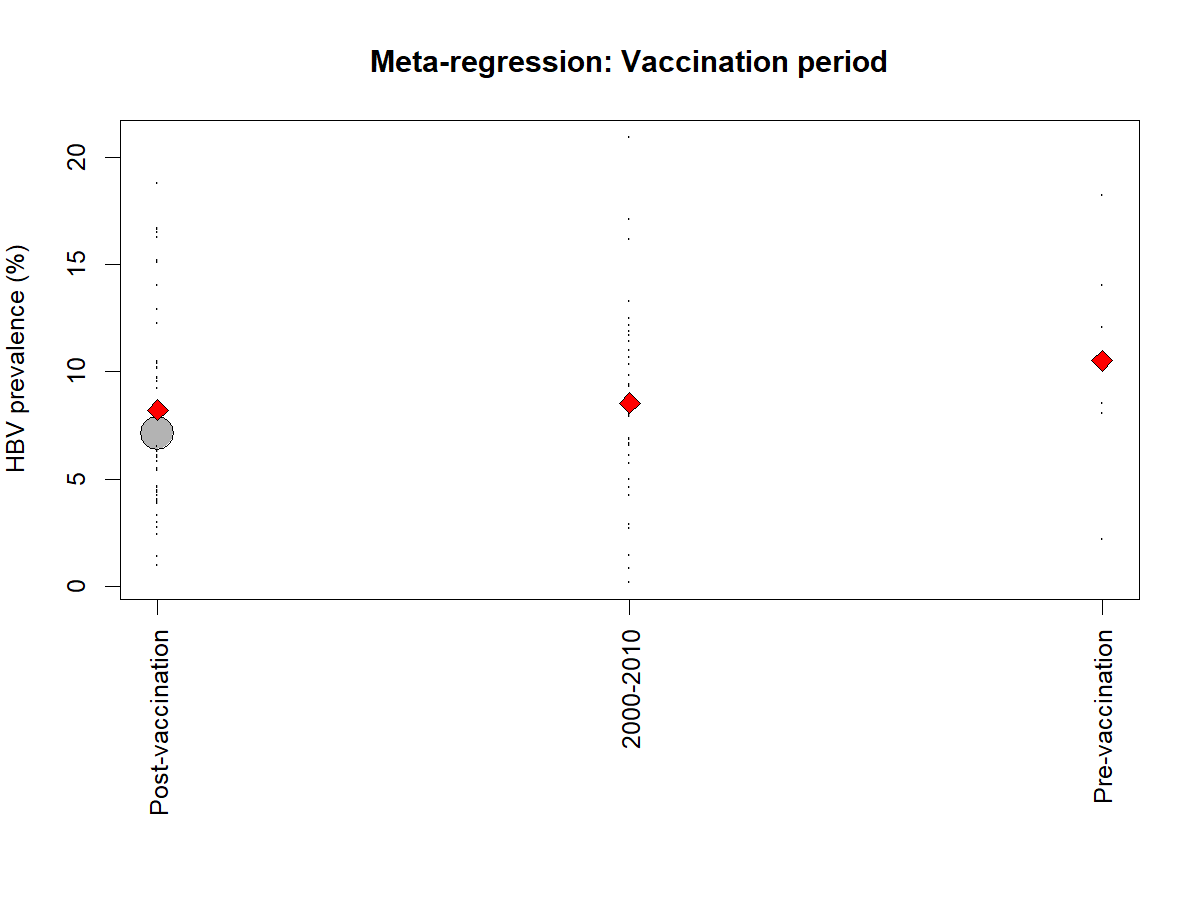

Supplement: Supplementary file 6 — Supporting Information 6 Table S6: Meta‐regression analyses of prevalence of HBV in pregnant women in West Africa. [file BMRI-2026-3975525-s007.docx]
