# Supplementary material for: Hepatitis B Virus Infection and Risk Factors Among Pregnant Women in Healthcare Facilities in West Africa: A Systematic Review and Meta‐Analysis
Source: Biomed Res Int. 2026 Mar 24;2026:3975525. doi: 10.1155/bmri/3975525 (PMC13140436; doi:10.1155/bmri/3975525)
Supplement: Supplementary file 7 — Supporting Information 7 Table S7: Genotypes of HBV in pregnant women in West Africa. [file BMRI-2026-3975525-s006.docx]

S7 Table: Genotypes of HBV in pregnant women in West Africa

| Authors | Countries | Study period | Genotype | Number with HBV | Number of genotypes |
| --- | --- | --- | --- | --- | --- |
| Anabire et al., 2023 [1] | Ghana | 2016-2017 | E | 191 | 175 |
|  | Ghana | 2016-2017 | A and E | 191 | 9 |
|  | Ghana | 2016-2017 | D and E | 191 | 5 |
|  | Ghana | 2016-2017 | A, D and E | 191 | 2 |
| Candotti et al., 2007 [2] | Ghana | Not specified | E | 70 | 69 |
|  | Ghana | Not specified | A1 | 70 | 1 |
| De Paschale et al., 2014 [3] | Nigeria | Jul/2001-Sep/2001 | E | 44 | 19 |
| Falaye et al., 2014 [4] | Nigeria | Sep/2012-Jun/2013 | E | 7 | 5 |
| Faleye et al., 2014 [5] | Nigeria | 2012-2013 | E | 7 | 5 |
| Ouoba et al., 2023 [6] | Burkina Faso | Feb/2021- Nov/2021 | E | 63 | 37 |
|  | Burkina Faso | Feb/2021- Nov/2021 | A | 63 | 23 |

1. Anabire, N.G., O. Quaye, and G.K. Helegbe, *Circulation of multiple hepatitis B virus genotypes in individual pregnant women seeking antenatal care in northern Ghana.* Virology journal, 2023. **20**(1): p. 149.

2. Candotti, D., K. Danso, and J.-P. Allain, *Maternofetal transmission of hepatitis B virus genotype E in Ghana, west Africa.* The Journal of general virology, 2007. **88**(Pt 10): p. 2686-2695.

3. De Paschale, M., et al., *Prevalence of HBV, HDV, HCV, and HIV infection during pregnancy in northern Benin.* Journal of medical virology, 2014. **86**(8): p. 1281-7.

4. Faleye, T.O.C., et al., *Detection of hepatitis B virus isolates with mutations associated with immune escape mutants among pregnant women in Ibadan, southwestern Nigeria.* SpringerPlus, 2015. **4**: p. 43.

5. Faleye, T.O.C., et al., *Molecular epidemiology of hepatitis B virus among pregnant women in southwestern Nigeria.* International Journal of Infectious Diseases, 2014. **21**(SUPPL. 1): p. 332.

6. Ouoba, S., et al., *Intermediate hepatitis B virus infection prevalence among 1622 pregnant women in rural Burkina Faso and implications for mother-to-child transmission.* Scientific reports, 2023. **13**(1): p. 6115.
